# Synthesis and Biological Evaluation of Quinoline Derivatives as a Novel Class of Broad-Spectrum Antibacterial Agents

**DOI:** 10.3390/molecules24030548

**Published:** 2019-02-02

**Authors:** Hai-Gen Fu, Zhi-Wen Li, Xin-Xin Hu, Shu-Yi Si, Xue-Fu You, Sheng Tang, Yan-Xiang Wang, Dan-Qing Song

**Affiliations:** Beijing Key Laboratory of Antimicrobial Agents, Institute of Medicinal Biotechnology, Chinese Academy of Medical Sciences and Peking Union Medical College, Beijing 100050, China; h.g.fu@rug.nl (H.-G.F.); zhiwenli2012@126.com (Z.-W.L.); huxinxin1985@163.com (X.-X.H.); sisyimb@hotmail.com (S.-Y.S.); 13311123098@163.com (X.-F.Y.); songdanqingsdq@hotmail.com (D.-Q.S.)

**Keywords:** quinoline derivatives, antibacterial agents, structure-activity relationship, LptA, Mannich reaction

## Abstract

Nineteen new quinoline derivatives were prepared via the Mannich reaction and evaluated for their antibacterial activities against both Gram-positive (G^+^) and Gram-negative (G^−^) bacteria, taking compound **1** as the lead. Among the target compounds, quinolone coupled hybrid **5d** exerted the potential effect against most of the tested G^+^ and G^−^ strains with MIC values of 0.125–8 μg/mL, much better than those of **1**. Molecular-docking assay showed that compound **5d** might target both bacterial LptA and Top IV proteins, thereby displaying a broad-spectrum antibacterial effect. This hybridization strategy was an efficient way to promote the antibacterial activity of this kind, and compound **5d** was selected for the further investigation, with an advantage of a dual-target mechanism of action.

## 1. Introduction

Infections caused by multidrug-resistance (MDR) bacteria, especially “ESKAPE” pathogens [1,2,3,4], kill thousands of people worldwide per year, posing a greater health crisis to human beings [5,6]. However, we are losing the battle against never-ending resistance due to the limits of efficacy and life-span of current antibiotics [7,8], which makes drifting back to pre-antibiotic era possible. Therefore, there is an urgent need to develop broad-spectrum antibacterial candidates with novel chemical scaffold against both Gram-positive (G^+^) and Gram-negative (G^−^) bacteria for the treatment of bacterial infections, especially drug-resistant strains without cross-resistance to currently used drugs.

**Figure 1:**
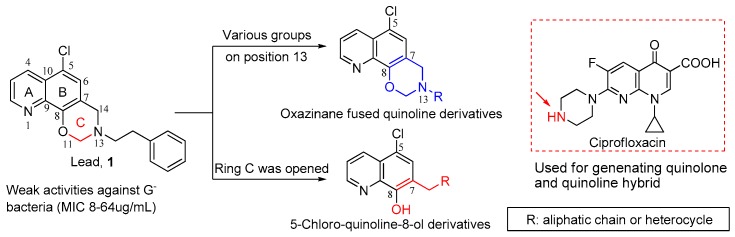
Chemical structure of compound **1** and structural modification strategies.

It is first reported that compound 5-chloro-13-phenethyl-13,14-dihydro-2*H*-[1,3]oxazino[5,6-h] quinoline (**1**, Figure 1) with a unique scaffold exhibited the weak antibacterial potencies against G^−^ bacteria including *Escherichia coli* and *Pseudomonas aeruginosa* [9] with unknown mode of action. Recently, we have demonstrated that compound **1** displayed reasonable activities against both drug-susceptible and resistant G^−^ bacteria with MICs ranging from 8 to 64 μg/mL. Furthermore, we first elucidated its novel mechanism of action against bacteria, mainly through blocking lipopoly saccharide (LPS) transport (Lpt) A-LptC interaction by targeting LptA [10], thereby killing G^−^ bacteria.

The unique chemical scaffold and specific mechanism of compound **1** against G^−^ greatly encouraged us to further explore the structure-activity relationship (SAR) of its kind, aiming at obtaining the antibacterial agents against G^−^ bacteria. According to the molecular docking assay, the oxazino quinoline core could fit well in the active binding site of LptA protein, while N13 side chain is more flexible and modifiable. Based on this strategy, SAR analysis was initially focused on the substituents at the N13-position. Therefore, taking **1** as the lead, phenylethyl at the 13-position was respectively replaced with various aliphatic chain and heterocycle, by which a series of oxazino quinoline derivatives (**4a**–**m**) were designed and prepared as shown in Figure 1. Alternatively, 5-chloro-quinoline-8-ol scaffold was reported to possess moderate activity against G^−^ bacteria [11]. Thus, ring C of compound **1** was opened, and another series of quinoline derivatives with 5-chloro atom (**5a**–**c**, **6a**, and **6b**) were constructed. Meanwhile, as described in Figure 1, quinolone fragment as a privileged substituent was linked with quinoline core to generate a hybrid compound **5d**, aiming at exploring dual-target candidate against both G^+^ and G^−^ bacteria. Thus, in the present study, 20 new derivatives of **1** were synthesized and evaluated for their in vitro antibacterial activities against both G^+^ and G^−^ bacteria using phenotype screening assay, and preliminary mechanism of action of the representative compounds were conducted as well.

## 2. Results and Discussion

### 2.1. Chemistry

**Scheme 1:**
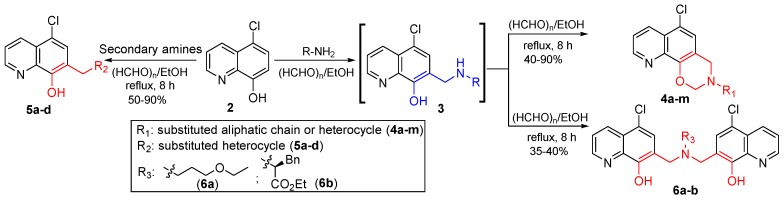
Synthetic route of all the target compounds.

All the target compounds were easily synthesized via the Mannich reaction of paraformaldehyde and various primary or secondary amines, using commercially available 5-chloro-8-hydroxyquinoline (**2**) as the starting material, as depicted in Scheme 1. The oxazino quinoline products (**4a**–**m**) were prepared via two continuous intermolecular- and intramolecular Mannich reaction in 40–90% yields using paraformaldehyde as the source of one-carbon unit, while compound **3** might be formed as a key transition-state intermediate. Similarly, when the primary amine was 3-ethoxypropan-1-amine or (S)-ethyl 2-amino-3-phenylpropanoate, the di-quinoline derivatives **6a** and **6b** were acquired instead in 35–40% yields, via two continuous intermolecular Mannich reaction. In addition, 5-chloro-quinoline-8-ol derivatives (**5a**–**d**) were also gained by a one-step Mannich reaction of **2**, paraformaldehyde and the corresponding secondary amines in yields of 50–90%.

### 2.2. Pharmacological evaluation

#### 2.2.1. SAR for Anti-Bacterial Activity

Totally 20 novel target compounds were screened on both G^+^ and G^−^ bacteria strains, and their structures and activity were listed in Table 1 and Table 2, respectively. Minimum inhibitory concentrations (MICs) assay were determined by the agar dilution method described by the Clinical Laboratory Standards Institute [12] with ciprofloxacin (CPFX) and lead **1** as the references. Bacteria strains used in this study were from the ATCC collection and clinical isolates in Chinese hospitals.

SAR analysis against G^+^ was first conducted as described in Table 1, taking **1** as the positive control. Firstly, the parent core (**2**) was tested, but it showed comparable potencies with that of **1**. Then, phenylethyl on 13-position of compound **1** was respectively replaced with various substituents, such as the aliphatic chain and heterocycle, by which a series of new oxazino quinoline derivatives (**4a**–**m**) were prepared and tested. However, most of them displayed weak activities against *Staphylococcus epidermidis*, *S. aureus*, *Enterococci faecalis* and *E. faecium* strains, except **4g**, **4h** with MIC values of 8–16 μg/mL, similar to that of **1**. Next, several quinoline derivatives (**5a**–**c**, **6a**–**b**) with different aliphatic chain and heterocycle at the 7-position were designed and measured. All of them had no activity against all the tested strains. Finally, quinolone as a privileged fragment was introduced on the 7-position of quinoline core, with which hybrid compound **5d** was constructed. As shown in Table 1, compound **5d** displayed potent effect against both susceptible and drug-resistant strains such as MRSA and VRE with the MIC values of 4–16 μg/mL, much greater than that of **1**.

Then, top three compounds **4g**, **4h**, and **5d** were chosen as the representative compounds to carry out the anti-G^−^ activity assay. As described in Table 2, lead **1** displayed weak activities against various G^−^ bacteria as reported [9], while compound **5d** exhibited a satisfactory result with MIC values of 0.125–8 μg/mL against some strains such as Escherichia coli, *Pseudomonas aeruginosa* and *Klebsiella pneumoniae* strains, much more active than those of **1** but less active than parent CPFX. The results indicated that compound **5d**, a hybrid with a quinolone skeleton, showed an exciting promise in both anti-G^+^ and G^−^ bacteria. Thus, we speculated that the introduction of a quinolone skeleton might be an effective way to modify this kind of compounds into a novel class of broad-spectrum antibacterial agents with a specific mode of action.

#### 2.2.2. Molecular-Docking Assay on Compound **5d**

**Figure 2:**
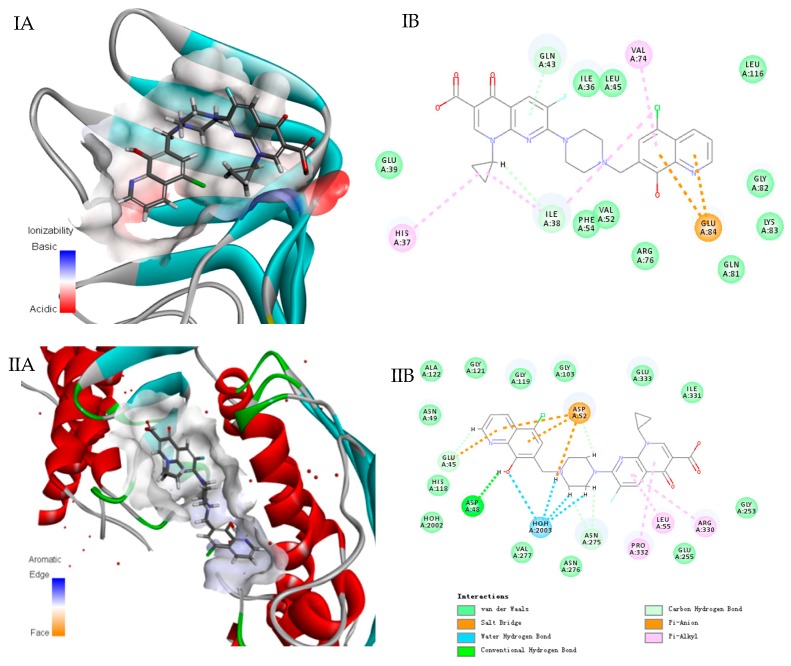
Interactions of compound **5d** in the active site of the LptA and DNA Top IV (Docking with Discovery Studio). (I A/B) LptA; (II A/B) Top IV. Compound is shown as stick models with green carbon atoms, blue nitrogen atoms, red oxygen atoms, and yellow sulfur atoms. Hydrogen bonds are depicted as red dashes in three-dimensional view and green dashes in two-dimensional view. Salt bridge interactions are depicted as brown dashes; pi-sulfur interactions are depicted as yellow dashes; pi-alkyl interactions are depicted as purple dashes.

In order to further explore mechanism of action of compound **5d**, molecular-docking assay was conducted with the Discovery Studio 4.5 docking program (Edition 4.5; BIOVIA: San Diego, CA, USA, 2015). Owing to promising potencies against G^−^ bacteria, the docking assay between LptA protein and **5d** interactions was first carried out. As depicted in Figure 2, compound **5d** fits well in the active hydrophobic pocket of the binding site (Figure 2 IA, brown area), and van der Waals forces, Pi-anion, and hydrophobic interaction (Figure 2 IB) contributed together to the interactions. The results suggested that LptA protein might be one of the targets which gave an explanation why **5d** exerted potential potencies against G^−^ bacteria.

Additionally, based on the quinolone fragment in compound **5d**, interactions between quinolone binding site (DNA Top IV) and **5d** were calculated. As expected, it also fits well in the active hydrophobic pocket of the binding site (Figure 2 IIA, brown area), and hydrogen bond with ASP48, van der Waals forces, water-hydrogen bonds with HOH2003 and hydrophobic interaction (Figure 2 IIB) might exist strong interactions. Thus, the docking results indicated that **5d** might own a dual-target mechanism of action for LptA and Top IV, thereby showing broad-spectrum antibacterial activity against both G^+^ and G^−^ bacteria. In addition, the docking assay between other target compounds and LptA/Top IV were also conducted, respectively (data not shown), less interactions were predicted due to the absence of quinolone fragment which is consistent with the phenotype screening results. Therefore, the probable mechanism of hybrid compound **5d** against bacteria was that the hydroxyquinoline fragment might be responsible for LptA interaction, while the quinolone moiety might inhibit Top IV protein activity, respectively.

## 3. Experimental Section

### 3.1. Apparatus, Materials, and Analysis Reagents

Melting point (mp) was obtained with CXM-300 melting point apparatus and uncorrected. The ^1^H-NMR spectra was performed on a Varian Inova 500 or 600 MHz spectrometer (Varian, San Francisco, CA, USA) and ^13^C-NMR on a Bruker Avance III 400, 500, or 600 spectrometer (Bruker, Zürich, Switzerland) with Me_4_Si as the internal standard, all the samples were dissolved in DMSO-*d*_6_ before testing. Optical rotations were acquired in Tetrahydrofuran (THF) using Autopol IV automatic polarimeter (Rudolph Research Analytical, Hackettstown, New Jersey, USA). High-resolution mass spectra (HRMS-ESI) data was recorded on an Autospec UItima-TOF mass spectrometer (Micromass UK Ltd., Manchester, UK). Flash chromatography was performed on CombiflashRf 200 (Teledyne, Lincoln, NE, USA), particle size 0.038 mm.

### 3.2. Chemistry

#### General Procedure for the Synthesis of Compounds **4a–m**, **5a–d**, **6a**, and **6b**

To a stirred solution of 5-chloroquinolin-8-ol (**2**, 360 mg, 2.0 mmol) in dry EtOH (10 mL) was added appropriate amines (2.2 mmol) and paraformaldehyde (240 mg, 8.0 mmol). The reaction mixture was heated to reflux for 8 h under nitrogen atmosphere. After completion of the reaction, the reaction mixture was allowed to cool down to room temperature and then kept in ice-bath for 3 h. The product was precipitated from the reaction mixture. The desired product was filtered off, washed with cold EtOH (3 mL) and dried under vacuum overnight.

*5-Chloro-13-methyl-3, 4-dihydro-2H-[1,3]oxazino-[5,6-h]quinoline* (**4a**). Light yellow solid. 351 mg (75% yield). Mp: 108–109 °C. ^1^H-NMR (500 MHz, DMSO-*d*_6_) δ 8.91 (dd, *J* = 4.1, 1.6 Hz, 1H), 8.44 (dd, *J* = 8.5, 1.6 Hz, 1H), 7.65 (dd, *J* = 8.5, 4.1 Hz, 1H), 7.46 (s, 1H), 4.99 (s, 2H), 4.05 (s, 2H), 2.54 (s, 3H); ^13^C-NMR (126 MHz, CDCl_3_) δ 149.9, 148.4, 140.0, 133.0, 125.8 (2C), 121.9, 121.8, 117.9, 84.9, 51.9, 40.2. HRMS: calcd for C_12_H_11_N_2_OCl [M + H]^+^: 235.0632, found: 235.0633.

*5-Chloro-13-propyl-3, 4-dihydro-2H-[1,3]oxazino-[5,6-h]quinoline* (**4b**). Light yellow solid. 367 mg (75% yield). Mp: 125–126 °C. ^1^H-NMR (500 MHz, DMSO-*d*_6_) δ 8.90 (dd, *J* = 4.1, 1.6 Hz, 1H), 8.44 (dd, *J* = 8.5, 1.6 Hz, 1H), 7.65 (dd, *J* = 8.5, 4.1 Hz, 1H), 7.47 (s, 1H), 5.06 (s, 2H), 4.11 (s, 2H), 2.67 (t, *J* = 8.5, 7.4 Hz, 2H), 1.62–1.50 (m, 2H), 0.88 (t, *J* = 7.4 Hz, 3H); ^13^C-NMR (126 MHz, DMSO-*d*_6_) δ 150.2, 149.4, 139.8, 132.6, 126.8, 125.3, 122.8, 120.2, 119.5, 83.3, 53.3, 49.5, 21.3, 12.0. HRMS: calcd for C_14_H_15_N_2_OCl [M + H]^+^: 263.0953, found: 263.0946.

*5-Chloro-13-isopropyl-3, 4-dihydro-2H-[1,3]oxazino-[5,6-h]quinoline* (**4c**). Light yellow solid. 350 mg (67% yield). Mp: 123–124 °C. ^1^H-NMR (400 MHz, CDCl_3_) δ 8.93 (dd, *J* = 4.2, 1.6 Hz, 1H), 8.47 (dd, *J* = 8.5, 1.6 Hz, 1H), 7.48 (dd, *J* = 8.5, 4.2 Hz, 1H), 7.25 (s, 1H), 5.25 (s, 2H), 4. (2)19 (s, 2H), 3.21 (dt, *J* = 12.9, 6.4 Hz, 1H), 1.19 (d, *J* = 6.4 Hz, 6H); ^13^C-NMR (101 MHz, CDCl_3_) δ 149.8, 148.9, 140.1, 132.9, 125.7, 125.4, 121.8, 121.4, 119.8, 81.7, 51.0, 47.1, 21.5 (2C). HRMS: calcd for C_14_H_15_N_2_OCl [M + H]^+^: 263.0946, found: 263.0944.

*5-Chloro-13-allyl-3, 4-dihydro-2H-[1,3]oxazino-[5,6-h]quinoline* (**4d**). Light yellow solid. 351 mg (80% yield). Mp: 108–109 °C. ^1^H-NMR (400 MHz, CDCl_3_) δ 8.97 (dd, *J* = 4.2, 1.6 Hz, 1H), 8.51 (dd, *J* = 8.5, 1.6 Hz, 1H), 7.53 (dd, *J* = 8.5, 4.2 Hz, 1H), 7.25 (s, 1H), 5.98–5.88 (m, 1H), 5.26–5.21 (m, 2H), 5.18 (s, 2H), 4.13 (s, 2H), 3.48 (dt, *J* = 6.3, 1.3 Hz, 2H); ^13^C-NMR (101 MHz, CDCl_3_) δ 150.0, 148.8, 139.9, 134.7, 133.3, 126.0, 125.9, 122.0, 121.9, 118.9, 118.2, 83.4, 55.0, 49.2. HRMS: calcd for C_14_H_13_N_2_OCl [M + H]^+^: 261.0789, found: 261.0789.

*5-Chloro-13-(prop-2-yn-1-yl)-3, 4-dihydro-2H-[1,3]oxazino-[5,6-h]quinoline* (**4e**). Yellow solid. 423 mg (82% yield). Mp: 126–127 °C. ^1^H-NMR (500 MHz, DMSO-*d*_6_) δ 8.91 (dd, *J* = 4.1, 1.6 Hz, 1H), 8.45 (dd, *J* = 8.5, 1.6 Hz, 1H), 7.66 (dd, *J* = 8.5, 4.1 Hz, 1H), 7.53 (s, 1H), 5.10 (s, 2H), 4.19 (s, 2H), 3.61 (d, *J* = 2.5 Hz, 2H), 3.27 (t, *J* = 2.5 Hz, 1H); ^13^C-NMR (126 MHz, DMSO-*d*_6_) δ 150.3, 149.0, 139.8, 132.6, 126.6, 125.4, 123.0, 120.7, 118.8, 82.0, 80.76, 76.0, 49.1, 40.8. HRMS: calcd for C_14_H_11_N_2_OCl [M+H]^+^: 259.0633, found: 259.0642.

*5-Chloro-13-(2-dimethylaminoethyl)-3, 4-dihydro-2H-[1,3]oxazino-[5,6-h]quinoline* (**4f**). Light yellow solid. 436 mg (75% yield). Mp: 105–106 °C. ^1^H-NMR (400 MHz, CDCl_3_) δ 8.95 (dd, *J* = 4.2, 1.7 Hz, 1H), 8.50 (dd, *J* = 8.5, 1.7 Hz, 1H), 7.52 (dd, *J* = 8.5, 4.2 Hz, 1H), 7.26 (s, 1H), 5.18 (s, 2H), 4.20 (s, 2H), 3.04 (t, *J* = 6.4 Hz, 2H), 2.65 (t, *J* = 6.4 Hz, 2H), 2.37 (s, 6H); ^13^C-NMR (101 MHz, CDCl_3_) δ 150.0, 148.8, 140.0, 133.1, 126.0, 125.9, 122.1, 121.9, 118.3, 83.7, 57.3, 50.4, 49.0, 45.4 (2C). HRMS: calcd for C_15_H_18_N_3_OCl [M + H]^+^: 292.1211, found: 292.1210.

*5-Chloro-13-cyanoethyl-3, 4-dihydro-2H-[1,3]oxazino[5,6-h]quinoline* (**4g**). White solid. 382 mg (70% yield). Mp: 188–189 °C. ^1^H-NMR (400 MHz, CDCl_3_) δ 8.96 (dd, *J* = 4.2, 1.6 Hz, 1H), 8.51 (dd, *J* = 8.5, 1.6 Hz, 1H), 7.54 (dd, *J* = 8.6, 4.2 Hz, 1H), 7.26 (s, 1H), 5.16 (s, 2H), 4.20 (s, 2H), 3.16 (t, *J* = 6.6 Hz, 2H), 2.63 (t, *J* = 6.6 Hz, 2H); ^13^C-NMR (101 MHz, CDCl_3_) δ 150.1, 148.6, 139.9, 133.1, 126.0, 125.5, 122.4, 122.2, 118.3, 117.8, 83.1, 50.4, 47.9, 17.9. HRMS: calcd for C_14_H_12_N_3_OCl [M + H]^+^: 274.0742, found: 274.0741.

*5-Chloro-13-methoxycarbonylmethyl-3,4-dihydro-2H-[1,3]oxazino[5,6-h]quinoline* (**4h**). Light yellow solid. 350 mg (60% yield). Mp: 112–113 °C. ^1^H-NMR (500 MHz, DMSO-*d*_6_) δ 8.91 (dd, *J* = 4.1, 1.6 Hz, 1H), 8.46 (dd, *J* = 8.5, 1.6 Hz, 1H), 7.66 (dd, *J* = 8.5, 4.1 Hz, 1H), 7.49 (s, 1H), 5.08 (s, 2H), 4.18 (s, 2H), 3.66 (s, 2H), 3.65 (s, 3H); ^13^C-NMR (126 MHz, DMSO-*d*_6_) δ 171.0, 150.3, 149.1, 139.9, 132.6, 126.8, 125.4, 123.0, 120.6, 119.1, 83.2, 52.9, 52.0, 50.0. HRMS: calcd for C_14_H_13_N_2_O_3_Cl [M + H]^+^: 293.0688, found: 293.0687.

*5-Chloro-13-benzoxycarbonylmethyl-3,4-dihydro-2H-[1,3]oxazino[5,6-h]quinoline* (**4i**). Yellow solid. 550 mg (75% yield). Mp: 111–112 °C. ^1^H-NMR (400 MHz, CDCl_3_) δ 8.95 (dd, *J* = 4.2, 1.6 Hz, 1H), 8.50 (dd, *J* = 8.5, 1.7 Hz, 1H), 7.52 (dd, *J* = 8.5, 4.1 Hz, 1H), 7.35–7.33 (m, 5H), 7.23 (s, 1H), 5.191 (s, 2H), 5.187 (s, 2H), 4.24 (s, 2H), 3.73 (s, 2H). ^13^C-NMR (101 MHz, CDCl_3_) δ 170.3, 150.1, 148.5, 139.9, 135.3, 133.1, 128.6, 128.6, 128.5, 128.4, 128.2, 126.0, 125.6, 122.3, 122.1, 117.6, 83.8, 66.8, 53.3, 50.6. HRMS: calcd for C_20_H_17_N_2_O_3_Cl [M + H]^+^: 369.1000, found: 369.1003.

*5-Chloro-13-cyclopropyl-3,4-dihydro-2H-[1,3]oxazino[5,6-h]quinoline* (**4j**). White solid. 312 mg (60% yield). Mp: 154–155 °C. ^1^H-NMR (400 MHz, CDCl_3_) δ 8.96 (dd, *J* = 4.2, 1.7 Hz, 1H), 8.51 (dd, *J* = 8.5, 1.7 Hz, 1H), 7.52 (dd, *J* = 8.5, 4.2 Hz, 1H), 7.29 (s, 1H), 5.18 (s, 2H), 4.21 (s, 2H), 2.51–2.45 (m, 1H), 0.62–0.57 (m, 4H). ^13^C-NMR (101 MHz, CDCl_3_) δ 149.9, 148.8, 140.0, 132.9, 125.8, 125.8, 121.9, 121.5, 118.6, 83.5, 50.8, 33.1, 7.2 (2C). HRMS: calcd for C_14_H_13_N_2_OCl [M + H]^+^: 261.0789, found: 261.0789.

*5-Chloro-13-[2-(pyrrolidin-1-yl)ethyl]-3,4-dihydro-2H-[1,3]oxazino[5,6-h]quinoline* (**4k**). Light yellow solid. 317 mg (60% yield). Mp: 135–136 °C. ^1^H-NMR (400 MHz, CDCl_3_) δ 8.94 (dd, *J* = 4.2, 1.7 Hz, 1H), 8.49 (dd, *J* = 8.5, 1.7 Hz, 1H), 7.51 (dd, *J* = 8.5, 4.2 Hz, 1H), 7.24 (s, 1H), 5.18 (s, 2H), 4.18 (s, 2H), 3.02 (t, *J* = 6.8 Hz, 2H), 2.73 (t, *J* = 6.8 Hz, 2H), 2.55 (t, *J* = 6.6 Hz, 4H), 1.80–1.78 (m, 4H); ^13^C-NMR (101 MHz, CDCl_3_) δ 150.0, 148.9, 140.0, 133.1, 125.9, 125.9, 122.0, 121.8, 118.4, 84.0, 54.7, 54.5 (2C), 50.6, 50.48, 23.5 (2C). HRMS: calcd for C_17_H_20_N_3_OCl [M + H]^+^: 318.1368, found: 318.13645.

*5-Chloro-13-[(tetrahydro-2H-pyran-4-yl)methyl]-3,4-dihydro-2H-[1,3]oxazino[5,6-h]quinoline* (**4l**). Light yellow solid. 512 mg (90% yield). Mp: 151–152 °C. ^1^H-NMR (400 MHz, CDCl_3_) δ 8.94 (dd, *J* = 4.2, 1.6 Hz, 1H), 8.48 (dd, *J* = 8.5, 1.7 Hz, 1H), 7.50 (dd, *J* = 8.5, 4.2 Hz, 1H), 7.24 (s, 1H), 5.13 (s, 2H), 4.10 (s, 2H), 3.98 (dd, *J* = 10.6, 3.5 Hz, 2H), 3.40 (td, *J* = 11.8, 2.0 Hz, 2H), 2.70 (d, *J* = 7.2 Hz, 2H), 1.86–1.80 (m, 1H), 1.71–1.67 (m, 2H), 1.35–1.25 (m, 2H); ^13^C-NMR (101 MHz, CDCl_3_) δ 150.0, 148.9, 139.9, 133.2, 125.9, 125.9, 122.1, 121.7, 118.4, 84.1, 67.9 (2C), 58.0, 50.9, 33.8 (2C), 31.3. HRMS: calcd for C_17_H_19_N_2_O_2_Cl [M + H]^+^: 319.1208, found: 319.1205.

*5-Chloro-13-morpholinopropyl-3,4-dihydro-2H-[1,3]oxazino[5,6-h]quinoline* (**4m**). Light yellow solid. 590 mg (85% yield). Mp: 124–125 °C. ^1^H-NMR (400 MHz, CDCl_3_) δ 8.94 (dd, *J* = 4.2, 1.7 Hz, 1H), 8.49 (dd, *J* = 8.5, 1.7 Hz, 1H), 7.51 (dd, *J* = 8.5, 4.2 Hz, 1H), 7.24 (s, 1H), 5.16 (s, 2H), 4.13 (s, 2H), 3.73–3.69 (m, 4H), 2.88 (t, *J* = 7.2 Hz, 2H), 2.44–2.39 (m, 6H), 1.83–1.77 (m, 2H); ^13^C-NMR (101 MHz, CDCl_3_) δ 150.0, 148.9, 140.0, 133.1, 125.9, 125.9, 122.0, 121.7, 118.3, 83.6, 67.0 (2C), 56.6, 53.8 (2C), 50.3, 49.8, 25.3. HRMS: calcd for C_18_H_22_N_3_O_2_Cl [M + H]^+^: 348.1473, found: 348.1471.

*5-Chloro-7-(pyrrolidin-1-yl-methyl)quinolin-8-ol* (**5a**). Light yellow solid. 341 mg (65% yield). Mp: 104–105 °C. ^1^H-NMR (400 MHz, CDCl_3_) δ 10.72 (br, 1H), 8.91 (dd, *J* = 4.2, 1.6 Hz, 1H), 8.46 (dd, *J* = 8.5, 1.6 Hz, 1H), 7.48 (dd, *J* = 8.5, 4.2 Hz, 1H), 7.33 (s, 1H), 3.98 (s, 2H), 2.73–2.69 (m, 4H), 1.90–1.87 (m, 4H); ^13^C-NMR (101 MHz, CDCl_3_) δ 152.6, 149.3, 139.8, 132.7, 127.0, 126.0, 121.9, 119.6, 119.1, 57.5, 53.7 (2C), 23.7 (2C). HRMS: calcd for C_14_H_15_N_2_OCl [M + H]^+^: 263.0946, found: 263.0945.

*5-Chloro-7-(morpholinomethyl)quinolin-8-ol* (**5b**). Light yellow solid. 361 mg (65% yield). Mp: 148–149 °C. Light yellow solid. ^1^H-NMR (400 MHz, CDCl_3_) δ 10.60 (br, 1H), 8.90 (dd, *J* = 4.2, 1.6 Hz, 1H), 8.47 (dd, *J* = 8.5, 1.6 Hz, 1H), 7.50 (dd, *J* = 8.5, 4.2 Hz, 1H), 7.40 (s, 1H), 3.84 (s, 2H), 3.78 (t, *J* = 4.8 Hz, 4H), 2.63 (t, *J* = 4.4 Hz, 4H); ^13^C-NMR (101 MHz, CDCl_3_) δ 151.7, 149.2, 139.5, 132.8, 127.6, 126.0, 122.1, 120.1, 118.0, 66.8 (2C), 59.6, 53.2 (2C). HRMS: calcd for C_14_H_15_N_2_O_2_Cl [M + H]^+^: 279.0895, found: 279.0896.

*5-Chloro-7-[(4-methylpiperazin-1-yl)methyl]quinolin-8-ol* (**5c**). Light yellow solid. 291 mg (50% yield). Mp: 109–110 °C. ^1^H-NMR (400 MHz, CDCl_3_) δ10.73 (br, 1H), 8.91 (dd, *J* = 4.2, 1.6 Hz, 1H), 8.46 (dd, *J* = 8.5, 1.6 Hz, 1H), 7.49 (dd, *J* = 8.5, 4.2 Hz, 1H), 7.33 (s, 1H), 3.86 (s, 2H), 2.68–2.48 (m, 8H), 2.31 (s, 3H); ^13^C-NMR (101 MHz, CDCl_3_) δ 152.4, 149.4, 139.8, 132.7, 127.3, 126.0, 122.0, 119.9, 118.1, 59.9, 54.9 (2C), 52.7 (2C), 45.9. HRMS: calcd for C_15_H_18_N_3_OCl [M + H]^+^: 292.1211, found: 292.1216.

*7-{4-[(5-Chloro-8-hydroxyquinolin-7-yl)methyl]piperazin-1-yl}-1-cyclopropyl-6-fluoro-4-oxo-1,4-dihydro-1,8-naphthyridine-3-carboxylic acid* (**5d**). White solid. 785 mg (75% yield). Mp: 242–243 °C. ^1^H-NMR (400 MHz, CDCl_3_) δ 14.96 (s, 1H), 10.41 (br, 1H), 8.91 (dd, *J* = 4.2, 1.5 Hz, 1H), 8.75 (s, 1H), 8.51 (dd, *J* = 8.5, 1.5 Hz, 1H), 7.55 (dd, *J* = 8.5, 4.2 Hz, 1H), 7.48 (s, 1H), 7.38 (d, *J* = 7.1 Hz, 1H), 3.96 (s, 2H), 3.55–3.51 (m, 1H), 3.42 (t, *J* = 4.8 Hz, 4H), 2.88 (t, *J* = 4.4 Hz, 4H), 1.42–1.37 (m, 2H), 1.21 (t, *J* = 4.9 Hz, 2H); ^13^C-NMR (101 MHz, CDCl_3_) δ 177.1, 166.9, 153.7 (d, *J* = 252.5 Hz), 151.3, 149.2, 147.5, 145.7 (d, *J* = 10.1 Hz), 139.2 (d, *J* = 36.4 Hz), 133.0, 127.8, 126.0, 122.3, 120.3, 120.10 (d, *J* = 8.1 Hz), 118.0, 112.5 (d, *J* = 23.2 Hz), 108.2, 105.1 (d, *J* = 3.0 Hz), 58.6, 52.4 (2C), 49.8 (2C), 35.31, 8.3 (2C). HRMS: calcd for C_26_H_24_N_5_O_4_ClF[M + H]^+^: 523.1543, found: 523.1545.

*7,7′-[[(3-Ethoxypropyl)azanediyl]bis(methylene)]bis(5-chloroquinolin-8-ol)* (**6a**). White solid. 340 mg (35% yield). Mp: 158–159 °C. ^1^H-NMR (400 MHz, CDCl_3_) δ 9.90 (br, 2H), 8.84 (dd, *J* = 4.1, 1.3 Hz, 2H), 8.43 (dd, *J* = 8.5, 1.3 Hz, 2H), 7.49–7.46 (m, 4H), 3.98 (s, 4H), 3.45 (t, *J* = 6.2 Hz, 2H), 3.36 (q, *J* = 7.0 Hz, 2H), 2.76 (t, *J* = 7.2 Hz, 2H), 2.03–1.92 (m, 2H), 1.00 (t, *J* = 7.0 Hz, 3H); ^13^C-NMR (101 MHz, CDCl_3_) δ 151.2 (2C), 148.9 (2C), 139.1 (2C), 132.9 (2C), 128.3 (2C), 125.9 (2C), 122.2 (2C), 120.1 (2C), 119.0 (2C), 68.2, 66.2, 54.4 (2C), 50.9, 26.7, 15.0. HRMS: calcd for C_25_H_25_N_3_O_3_Cl_2_ [M + H]^+^: 486.1346, found: 486.1347.

*(S)-Ethyl-2-[bis[(5-chloro-8-hydroxyquinolin-7-yl)methyl]amino]-3-phenylpropanoate* (**6b**). White solid. 460 mg (40% yield). [α]${}_{D}^{25}$–43.0 (c 0.10, THF); Mp: 170–171 °C. ^1^H-NMR (400 MHz, CDCl_3_) δ 8.80 (dd, *J* = 4.2, 1.5 Hz, 2H), 8.41 (dd, *J* = 8.5, 1.5 Hz, 2H), 7.48 (dd, *J* = 8.5, 4.2 Hz, 2H), 7.37 (s, 2H), 7.22–7.19 (m, 3H), 7.12–7.10 (m, 2H), 4.29–4.17 (m, 2H), 4.14 (s, 4H), 3.82 (dd, *J* = 8.1, 7.1 Hz, 1H), 3.33–3.16 (m, 2H), 1.30 (t, *J* = 7.1 Hz, 3H); ^13^C-NMR (101 MHz, CDCl3) δ 172.0, 149.9 (2C), 148.5 (2C), 138.6 (2C), 137.8, 133.1 (2C), 129.3, 128.8 (2C), 128.4 (2C), 126.6 (2C), 125.6 (2C), 122.1 (2C), 120.2 (2C), 120.2 (2C), 64.1, 60.8, 49.5 (2C), 35.2, 14.4. HRMS: calcd for C_31_H_27_N_2_O_4_Cl_2_ [M + H]^+^: 576.1451, found: 576.1454.

The ^1^H-NMR, and ^13^C-NMR data of compounds **4a**–**m**, **5a**–**d** and **6a**–**b** can obtain from the Supplementary Materials.

### 3.3. Antimicrobial Assay

Minimum inhibitory concentrations (MICs) of the 30 purified target compounds were determined by using the agar dilution assay at various concentrations of 128, 64.0, 32.0, 16.0, 8.0, 4.0, 2.0, 1.0, 0.5, 0.25, 0.125, 0.03, and 0.06 μg/mL described by the Clinical Laboratory Standards Institute as well as molar concentration (μM). Organisms used in this study included strains from the ATCC collection and clinical isolates from Chinese hospitals. CPFX were used as positive control drugs. The test medium was Mueller-Hinton broth, and the inoculum was 10,000 colony-forming units (cfu)/spot. Culture plates were incubated at 35 °C for 18 h, and MICs were then recorded. The MIC was defined as the lowest concentration that prevented visible growth of the bacteria.

## 4. Conclusions

In summary, thirteen oxazino quinoline and six quinoline derivatives were designed, prepared, and evaluated for their antibacterial activities against G^+^ and G^−^ strains taking **1** as the lead. Out of the newly synthesized target compounds, quinolone coupled hybrid **5d** exerted the promising effect with MIC values of 0.125–16 μg/mL against the most tested G^+^ and G^−^ bacteria. Molecular-docking assay showed that compound **5d** might target both bacterial LptA and Top IV proteins, thereby displaying a broad-spectrum activity against G^+^ and G^−^ organisms. The combination of a quinolone privileged skeleton and quinoline might be an efficient way to promote the antibacterial activity of this kind of compounds. This coupling strategy would offer powerful information on further strategic optimization of its kind, and compound **5d** was chosen for further investigation with an advantage of dual-target mechanism for LptA and Top IV.

## Figures and Tables

**Table 1:**
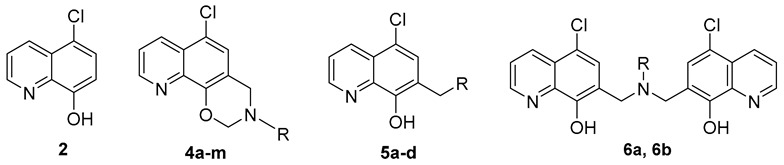
In vitro activities against G^+^ bacteria of the aimed compounds.

NO.	R	*Staphylococcus epidermidis* (MIC *^a^*)		*Staphylococcus.aureus* (MIC)				*Enterococci.faecalis* (MIC)		*Enterococci.faecium* (MIC)	
		ATCC 12228 MSSE *^b^*	12-6 MSSE	ATCC 29213 MSSA *^c^*	ATCC 33591 MRSA *^d^*	15 MSSA	12-28 MSSA	ATCC 29212 VSE *^e^*	ATCC 51299 VRE *^f^*	ATCC 700221 VRE	12-3 VSE
**1**	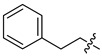	16 (49.4)	8 (24.7)	16 (49.4)	16 (49.4)	16 (49.4)	8 (24.7)	8 (24.7)	8 (24.7)	8 (24.7)	8 (24.7)
**2**	-	8 (44.7)	16 (89.4)	8 (44.7)	16 (89.4)	8 (44.7)	8 (44.7)	16 (89.4)	16 (89.4)	16 (89.4)	8 (44.7)
**4a**	CH_3_	32 (136)	32 (136)	32 (136)	32 (136)	32 (136)	32 (136)	32 (136)	32 (136)	32 (136)	32 (136)
**4b**	CH_3_CH_2_CH_2_-	32 (122)	32 (122)	32 (122)	32 (122)	32 (122)	32 (122)	32 (122)	32 (122)	32 (122)	32 (122)
**4c**	(CH_3_)_2_CH-	32 (122)	32 (122)	32 (122)	32 (122)	32 (122)	32 (122)	32 (122)	>64 (>243)	>64 (>243)	32 (122)
**4d**	CH_2_=CHCH_2_-	16 (61.3)	16 (61.3)	16 (61.3)	16 (61.3)	16 (61.3)	16 (61.3)	16 (61.3)	16 (61.3)	32 (123)	16 (61.3)
**4e**	CH≡CCH_2_-	16 (61.8)	16 (61.8)	32 (124)	16 (61.8)	32 (124)	32 (124)	16 (61.8)	16 (61.8)	16 (61.8)	16 (61.8)
**4f**	(CH_3_)_2_NCH_2_CH_2_-	32 (110)	32 (110)	32 (110)	32 (110)	32 (110)	64 (219)	32 (110)	32 (110)	64 (219)	64 (219)
**4g**	CNCH_2_CH_2_-	8 (29.2)	8 (29.2)	8 (29.2)	8 (29.2)	16 (58.4)	8 (29.2)	8 (29.2)	8 (29.2)	16 (58.4)	8 (29.2)
**4h**	CH_3_OCOCH_2_-	8 (27.3)	8 (27.3)	8 (27.3)	8 (27.3)	8 (27.3)	8 (27.3)	16 (54.6)	16 (54.6)	8 (27.3)	16 (54.6)
**4i**	PhCH_2_OCOCH_2_-	>64 (>173)	>64 (>173)	>64 (>173)	>64 (>173)	>64 (>173)	>64 (>173)	>64 (>173)	>64 (>173)	>64 (>173)	>64 (>173)
**4j**	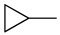	16 (61.3)	16 (61.3)	16 (61.3)	16 (61.3)	16 (61.3)	16 (61.3)	16 (61.3)	16 (61.3)	16 (61.3)	32 (123)
**4k**	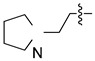	16 (50.3)	16 (50.3)	64 (201)	64 (201)	64 (201)	64 (201)	32 (101)	64 (201)	64 (201)	64 (201)
**4l**	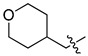	64 (201)	64 (201)	64 (201)	64 (201)	128 (401)	64 (201)	64 (201)	128 (401)	128 (401)	128 (401)
**4m**	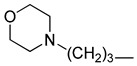	64 (184)	64 (184)	64 (184)	64 (184)	64 (184)	64 (184)	64 (184)	>64 (>184)	>64 (>184)	>64 (>184)
**5a**	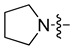	32 (122)	32 (122)	32 (122)	32 (122)	32 (122)	64 (243)	32 (122)	32 (122)	64 (243)	64 (243)
**5b**	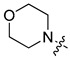	16 (57.3)	16 (57.3)	16 (57.3)	16 (57.3)	16 (57.3)	16 (57.3)	16 (57.3)	16 (57.3)	16 (57.3)	>128 (>459)
**5c**	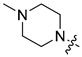	128 (438)	128 (438)	>128 (>438)	>128 (>438)	>128 (>438)	>128 (>438)	128 (438)	128 (438)	128 (438)	>128 (>438)
**5d**	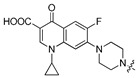	4 (7.65)	16 (30.6)	4 (7.65)	8 (15.3)	4 (7.65)	4 (7.65)	8 (7.65)	16 (15.3)	>128 (>245)	0.5 (0.956)
**6a**	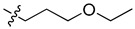	128 (263)	128 (263)	128 (263)	128 (263)	128 (263)	128 (263)	128 (263)	128 (263)	128 (263)	128 (263)
**6b**	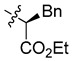	128 (222)	128 (222)	128 (222)	128 (222)	128 (222)	128 (222)	128 (222)	128 (222)	128 (222)	128 (222)
**CPFX**	-	0.125 (0.378)	0.5 (1.51)	0.25 (0.755)	0.25 (0.755)	0.125 (0.378)	0.25 (0.755)	0.5 (1.51)	0.5 (1.51)	>128 (>387)	64 (193)

*^a^* MIC was determined by agar dilution. MIC unit: μg/mL(μM). *^b^* MSSE, methicillin-sensitive *Staphylococcus epidermidis*; *^c^* MSSA, methicillin-sensitive *Staphylococcus aureus*; *^d^* MRSA, methicillin-resistant *Staphylococcus aureus*; *^e^* VSE, vancomycin-sensitive *Enterococci*; *^f^* VRE, vancomycin-resistant *Enterococci*.

**Table 2:** In vitro activities against G^−^ bacteria of candidates **4g**, **4h**, and **5d**.

Microorganism Strains No.	Phenotype	1 (MIC *^a^*)	2 (MIC)	4g (MIC)	4h (MIC)	5d (MIC)	CPFX (MIC)
*Escherichia coli* ATCC 25922	BL *^b^* (+)/ESBLs *^c^* (−)	32 (98.8)	16 (89.4)	64 (234)	>64 (>218)	0.25 (0.478)	≤0.03 (≤0.0906)
*E. coli*1515	BL(+)/ESBLs(−)	32 (98.8)	16 (89.4)	>64 (>234)	>64 (>218)	8 (15.3)	≤0.03 (≤0.0906)
*Klebsiella pneumoniae* ATCC 700603	ESBLs(+)	>128 (>395)	>64 (>358)	>64 (>234)	>64 (>218)	8 (15.3)	0.25 (0.755)
*K. pneumoniae*7	BL(+)/ESBLs(−)	>128 (>395)	>64 (>358)	>64 (>234)	>64 (>218)	0.5 (0.956)	≤0.03 (≤0.0906)
*K. pneumoniae*2146	NDM-1 *^d^* (+)	>128 (>395)	>128 (>715)	>64 (>234)	>64 (>218)	>128 (>245)	>128 (>388)
*K. pneumoniae*12-4	BL(+)/ESBLs(−)	128 (395)	>64 (>358)	>64 (>234)	>64 (>218)	1 (1.91)	≤0.03 (≤0.0906)
*K. pneumoniae*12-8	ESBLs(+)	128 (395)	>128 (>715)	>64 (>234)	>64 (>218)	>128 (>245)	16 (48.3)
*Pseudomonas aeruginosa* ATCC 27853	BL(+)	>128 (>395)	>128 (>715)	>64 (>234)	>64 (>218)	4 (7.65)	0.5 (1.51)
*P. aeruginosa* PA01	BL(+)	>128 (>395)	>128 (>715)	>64 (>234)	>64 (>218)	16 (30.6)	2 (6.04)
*P. aeruginosa*12-16	BL(+)	>128 (>395)	>128 (>715)	>64 (>234)	>64 (>218)	>128 (>245)	4 (12.1)
*Acinetobacter calcoacetious* ATCC 19606	BL(+)	128 (395)	64 (358)	32 (117)	64 (218)	16 (30.6)	0.5 (1.51)
*Enterobacter cloacae* ATCC 43560	BL(+)	128 (395)	64 (358)	>64 (>234)	>64 (>218)	8 (15.3)	≤0.03 (≤0.0906)
*Enterobacter aerogenes* ATCC 13048	BL(+)	128 (395)	128 (715)	>64 (>234)	>64 (>218)	>128 (>245)	≤0.03 (≤0.0906)
*Serratia marcescens* ATCC 21074	BL(+)	>128 (>395)	>128 (>715)	>64 (>234)	>64 (>218)	1 (1.91)	0.06 (0.181)
*Morganella morganii* ATCC 25830	BL(+)	>128 (>395)	64 (358)	>64 (>234)	>64 (>218)	0.5 (0.956)	≤0.03 (≤0.0906)
*Providentia rettgeri* ATCC 31052	BL(-)	>128 (>395)	16 (89.4)	64 (234)	>64 (>218)	0.125 (0.239)	≤0.03 (≤0.0906)
*Proteus vulgaris* ATCC 29905	BL(+)	128 (395)	64 (358)	64 (234)	>64 (>218)	0.125 (0.239)	≤0.03 (≤0.0906)
*Proteus mirabilis*12-6	BL(+)	>128 (>395)	32 (179)	>64 (>234)	>64 (>218)	64 (122)	2 (6.04)
*Stenotrophomonas maltoph.*ATCC 13636	BL(+)	32 (98.8)	64 (358)	64 (234)	>64 (>218)	128 (245)	2 (6.04)
*Citrobacter freundii* ATCC 43864	BL(+)	64 (198)	64 (358)	>64 (>234)	NT	0.5 (0.956)	≤0.03 (≤0.0906)

*^a^* MIC was determined by agar dilution. MIC unit: μg/mL(μM). *^b^* BL, Beta-lactamase; *^c^* ESBLs, Extended spectrum beta-lactamase; *^d^* NDM-1, New Delhi metallo-beta-lactamase 1.

## Supplementary Materials

The following are available online at http://www.mdpi.com/1420-3049/24/3/548/s1, the ^1^H-NMR, and ^13^C-NMR data of compounds **4a**–**m**, **5a**–**d** and **6a**–**b**.
