# Supplementary material for: Synthesis and Biological Evaluation of Quinoline Derivatives as a Novel Class of Broad-Spectrum Antibacterial Agents
Source: Molecules. 2019 Feb 2;24(3):548. doi: 10.3390/molecules24030548 (PMC6384568; doi:10.3390/molecules24030548)

Supporting Information

Synthesis and Biological evaluation of quinoline derivatives as a novel class of broad-spectrum antibacterial agents

Hai-Gen Fu, Zhi-Wen Li, Xin-Xin Hu, Xue-Lian Zhang, Shu-Yi Si, Xue-Fu You, Sheng Tang, Yan-Xiang Wang, Dan-Qing Song

*Figure S1. ^{1}H NMR (top) and ^{13}C NMR (bottom) of 5-Chloro-13-methyl-3, 4-dihydro-2H-[1, 3] oxazino-[5, 6-h]quinoline (**4a**)*

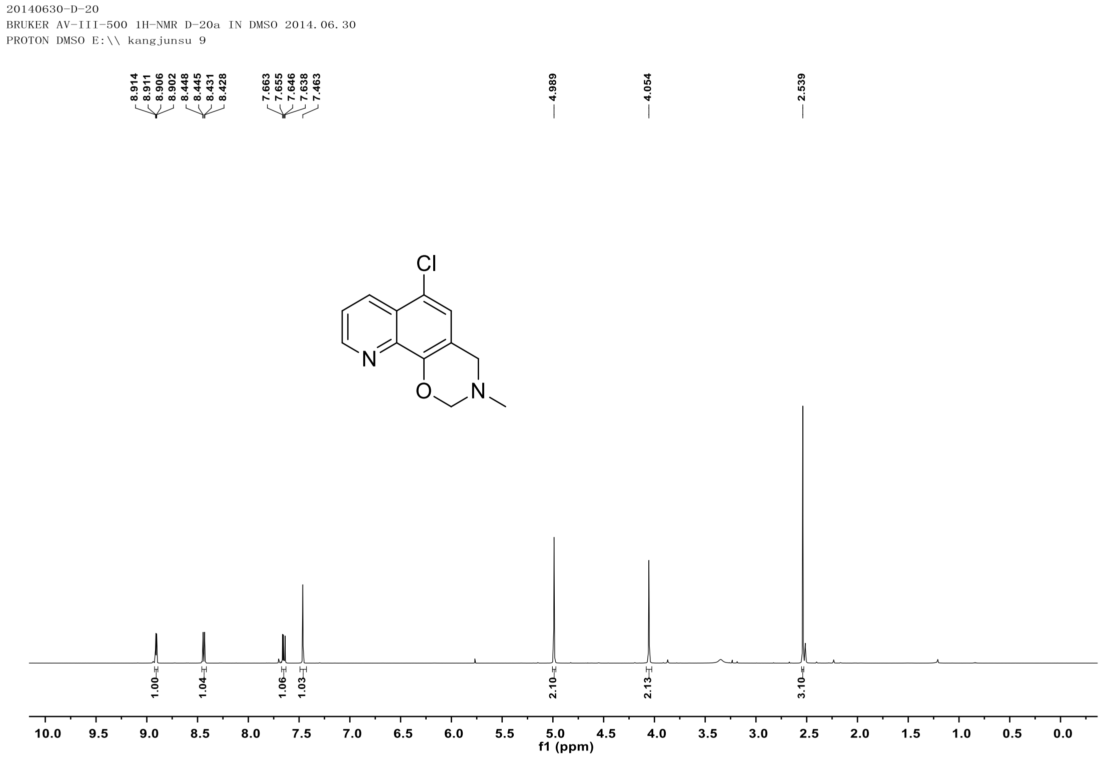

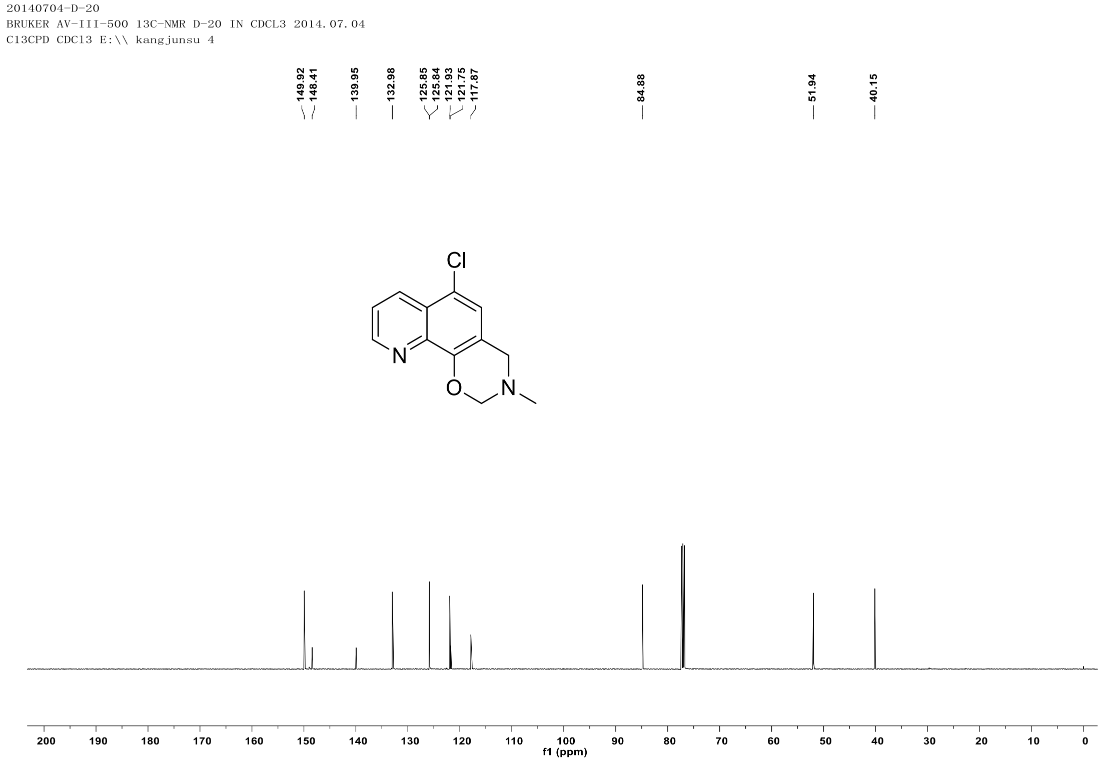

*Figure S2. ^{1}H NMR (top) and ^{13}C NMR (bottom) of 5-Chloro-13-propyl-3, 4-dihydro-2H-[1,3] oxazino-[5, 6-h]quinoline (**4b**)*

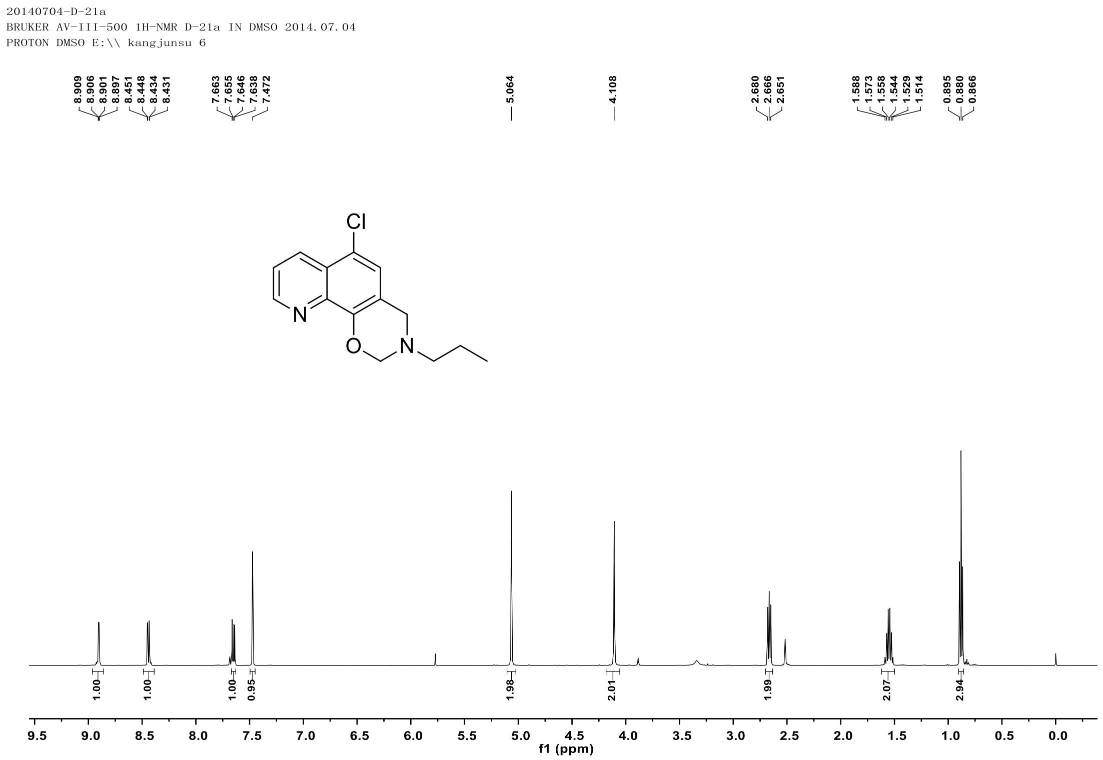

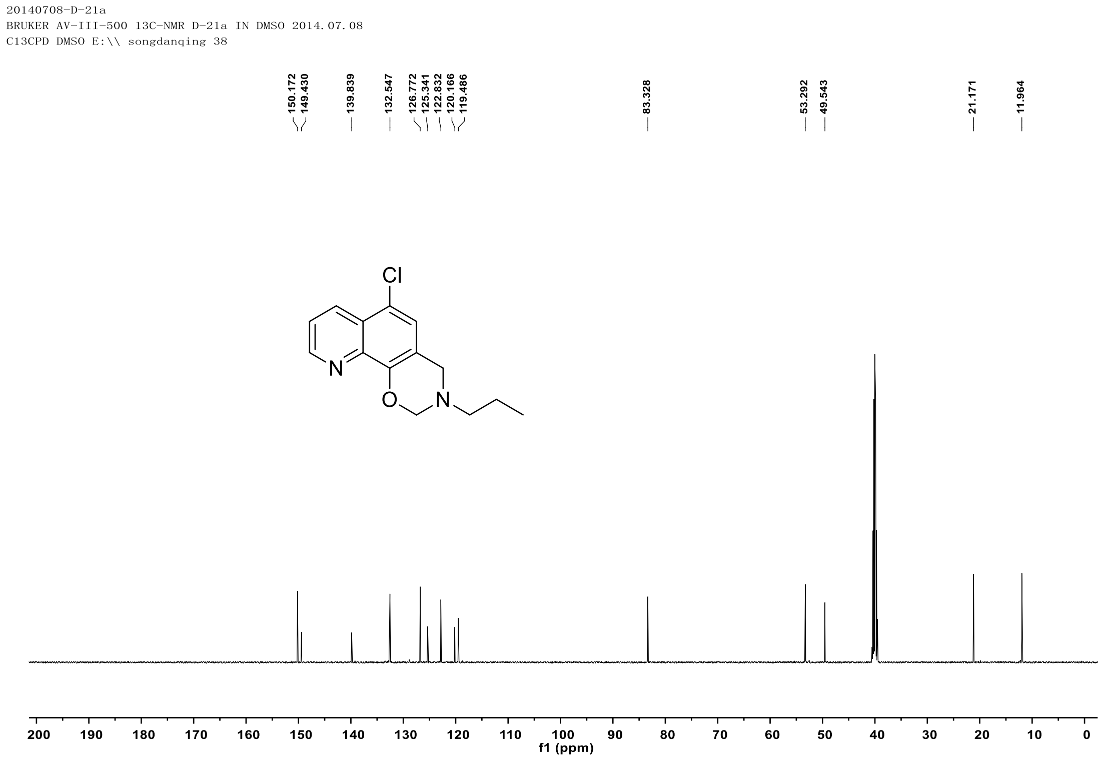

*Figure S3. ^{1}H NMR (top) and ^{13}C NMR (bottom) of 5-Chloro-13-isopropyl-3, 4-dihydro-2H-[1, 3] oxazino-[5, 6-h]quinoline (**4c**)*

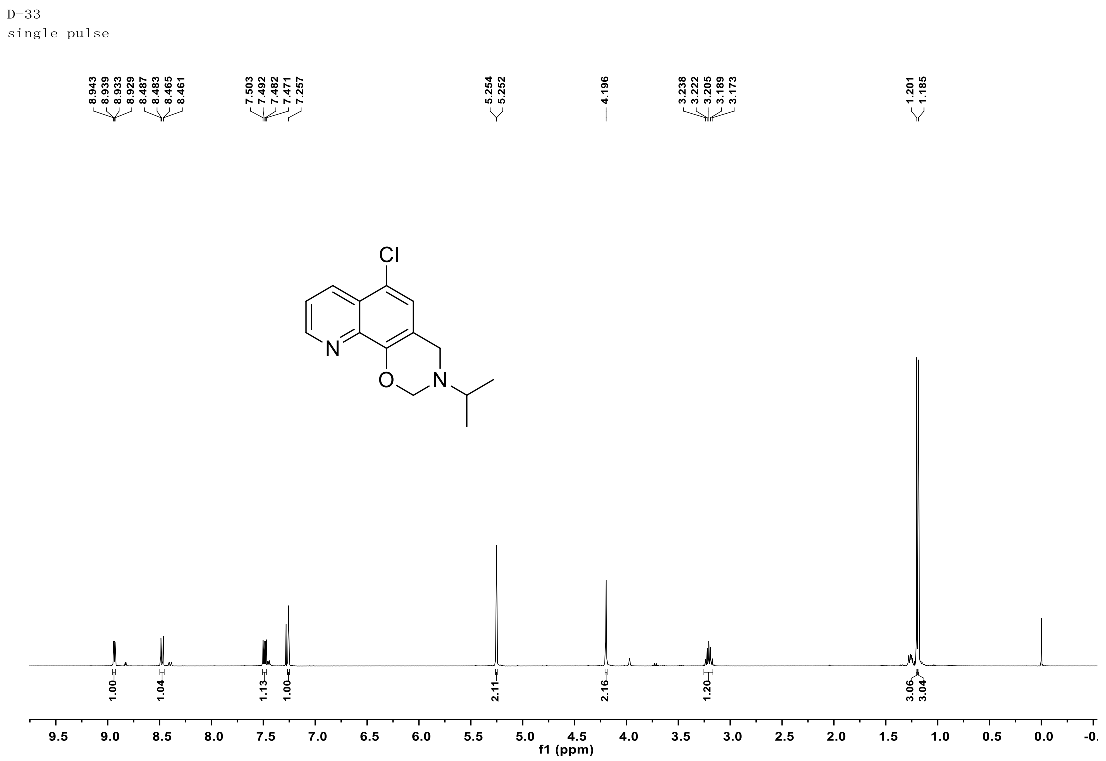

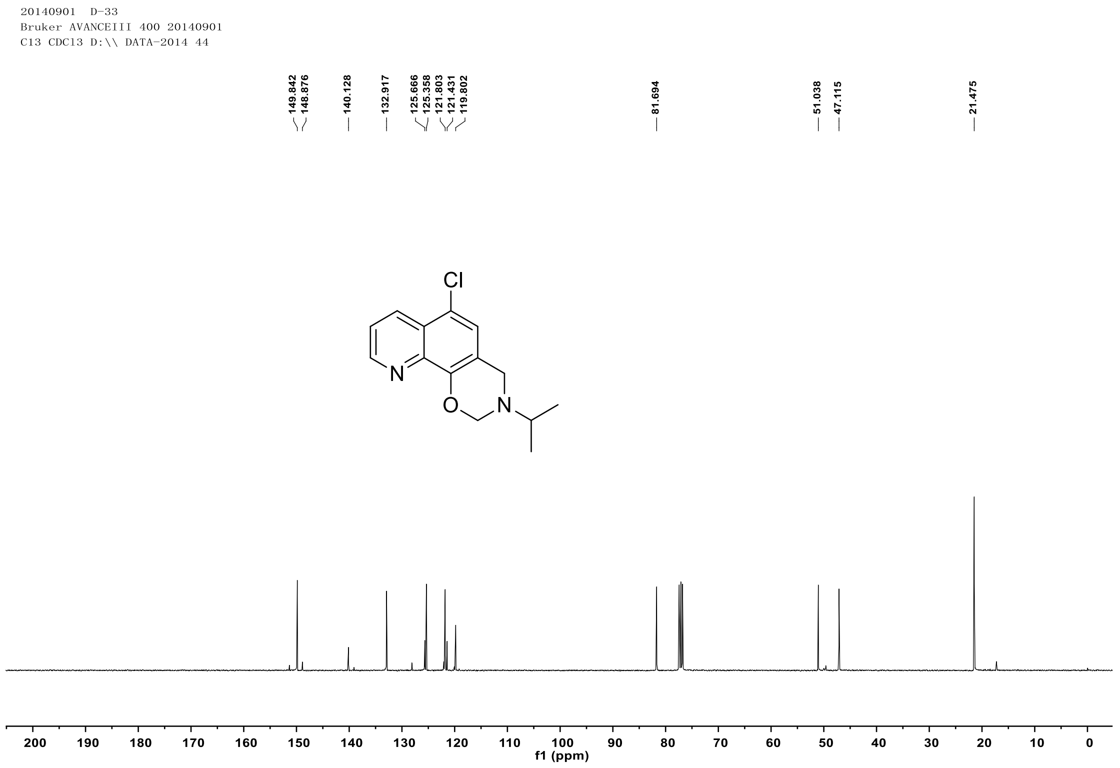

*Figure S4. ^{1}H NMR (top) and ^{13}C NMR (bottom) of 5-Chloro-13-allyl-3, 4-dihydro-2H-[1, 3] oxazino-[5, 6-h]quinoline (**4d**)*

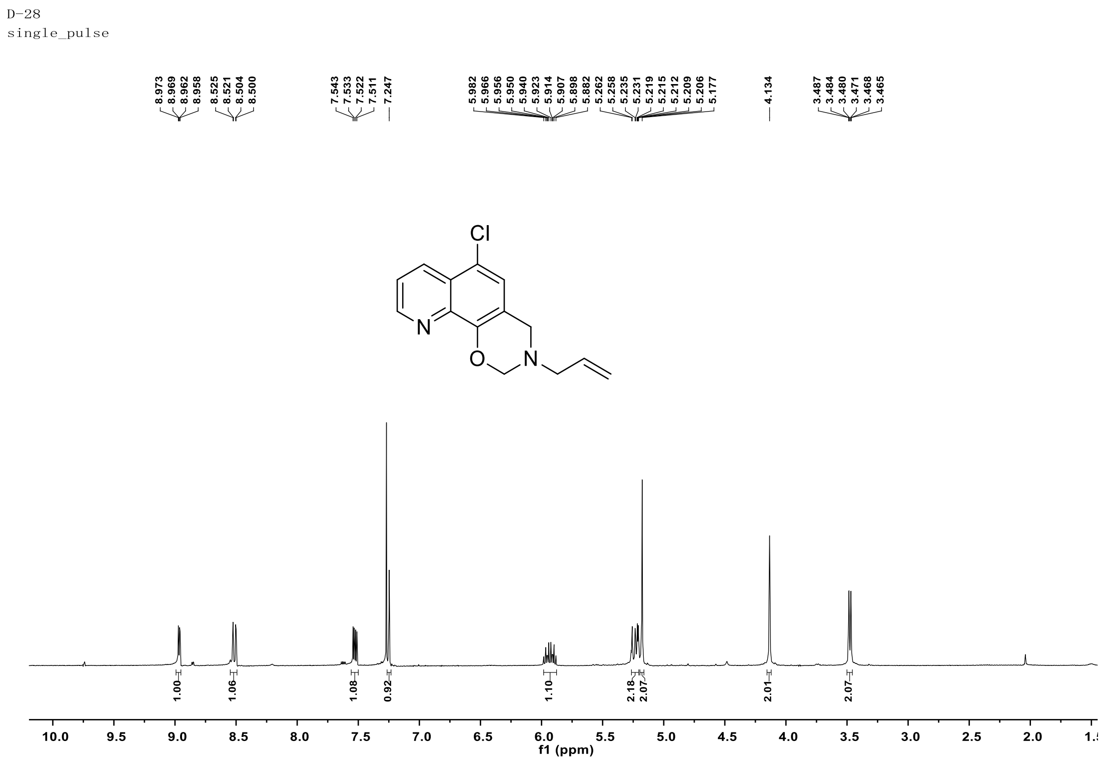

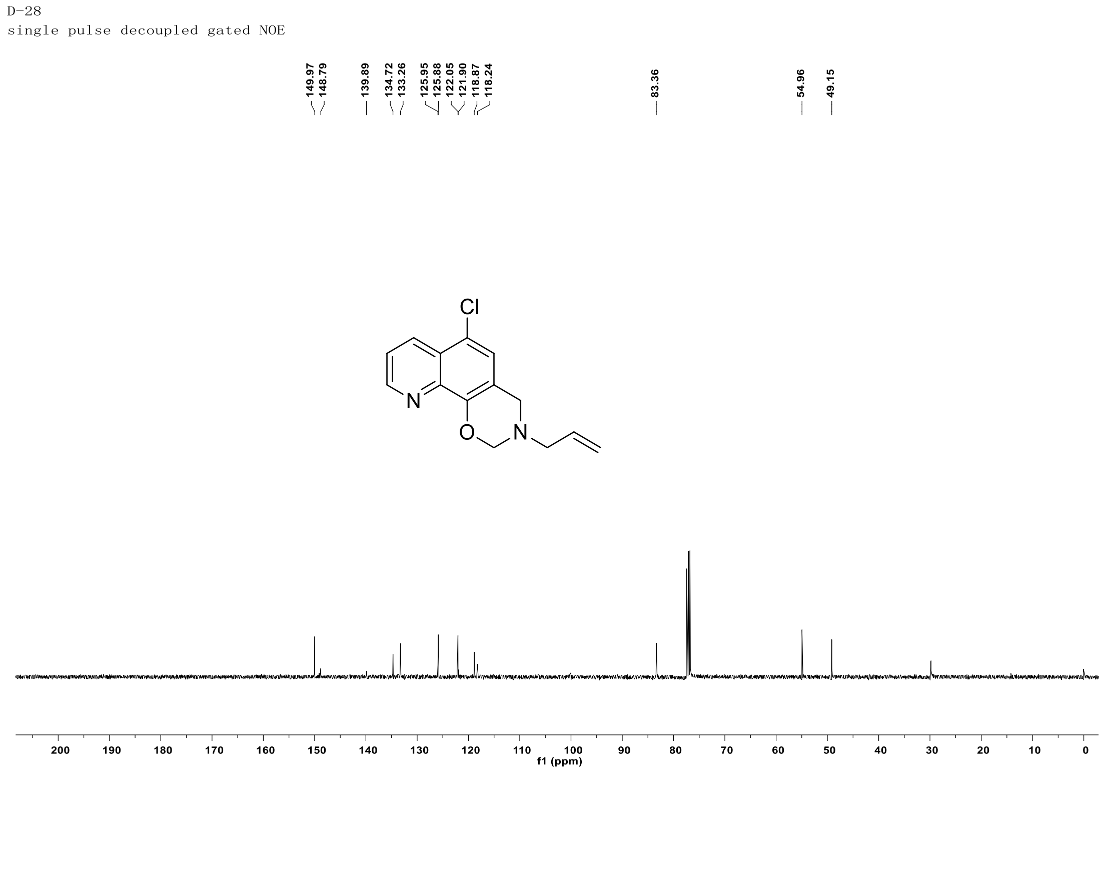

*Figure S5. ¹H NMR (top) and ¹³C NMR (bottom) of 5-Chloro-13-(prop-2-yn-1-yl)-3, 4-dihydro-2H-[1, 3] oxazino-[5, 6-h]quinoline (**4e**)*

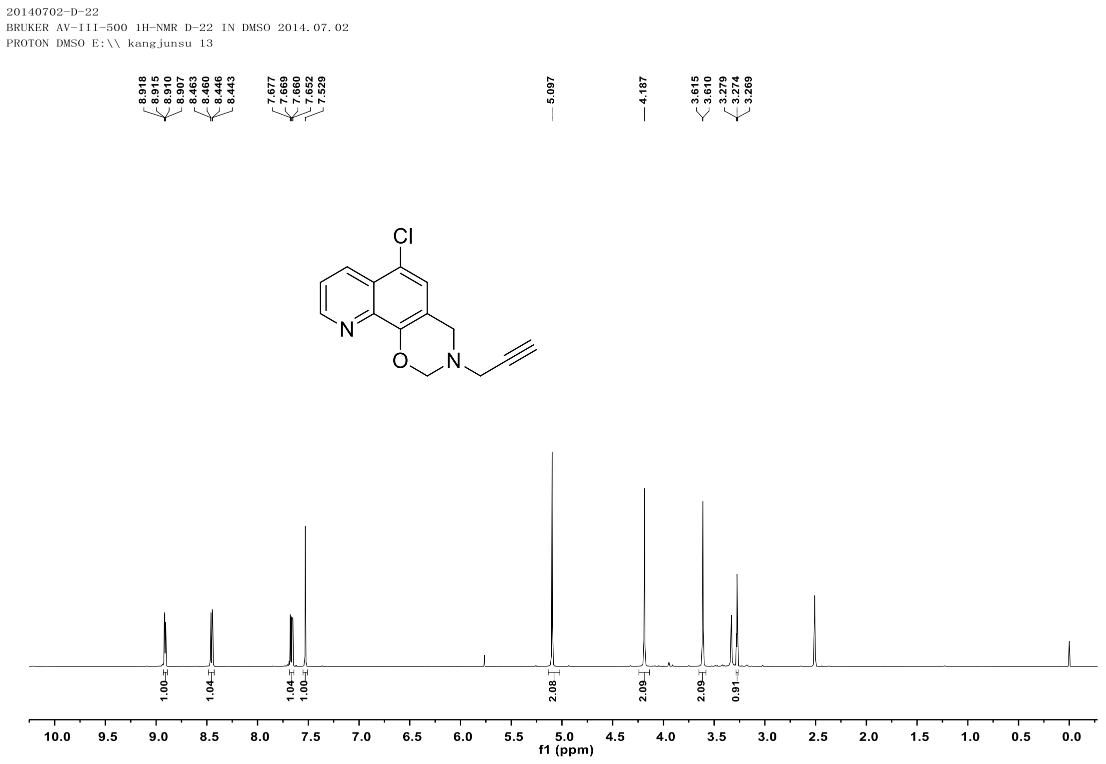

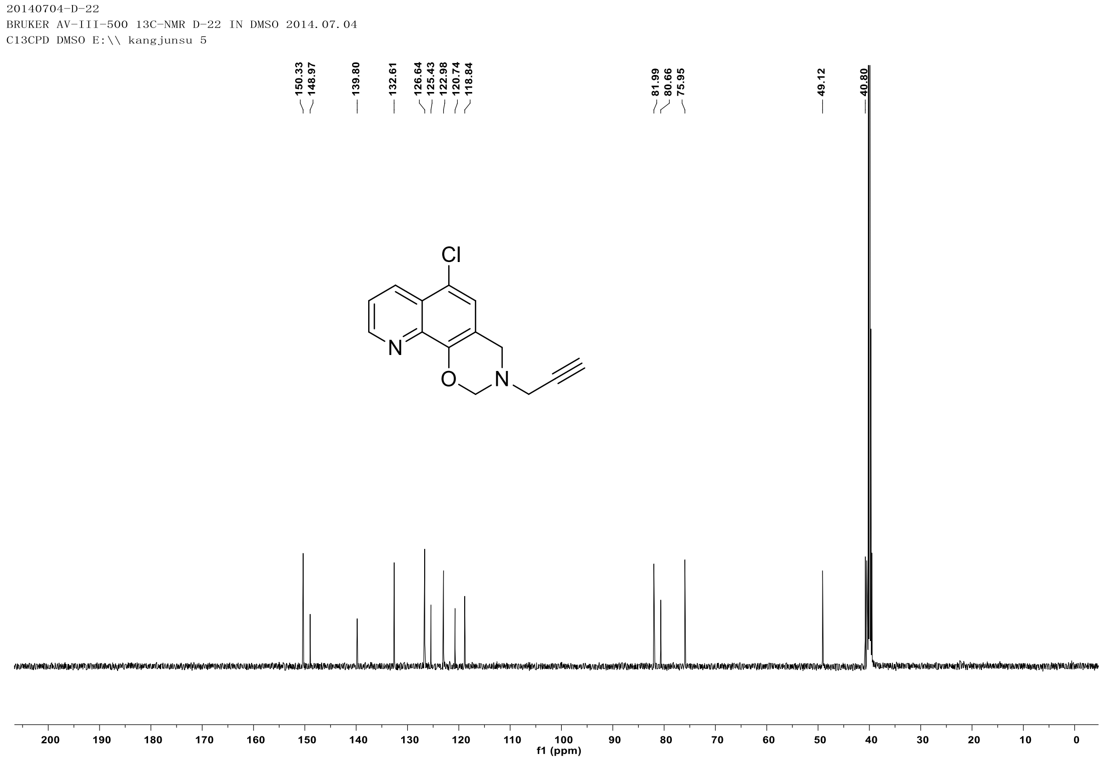

*Figure S6. ^{1}H NMR (top) and ^{13}C NMR (bottom) of 5-Chloro-13-(2-dimethylaminoethyl)-3, 4-dihydro-2H-[1, 3]oxazino-[5, 6-h]quinoline (**4f**)*

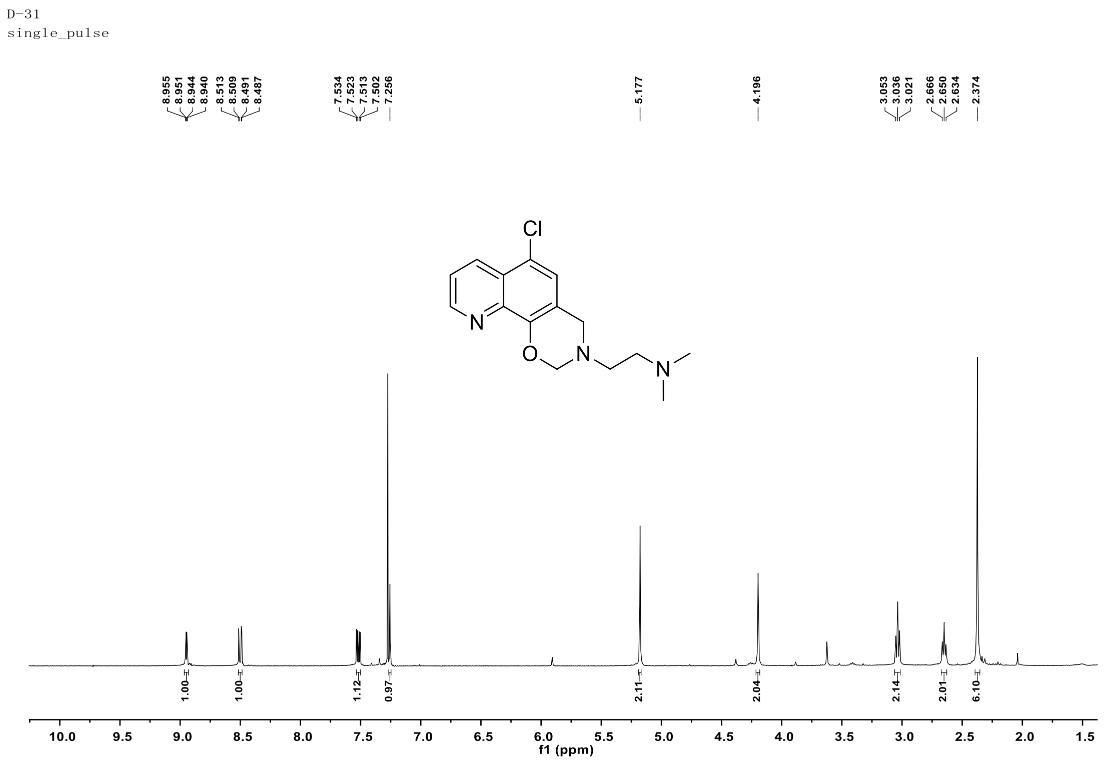

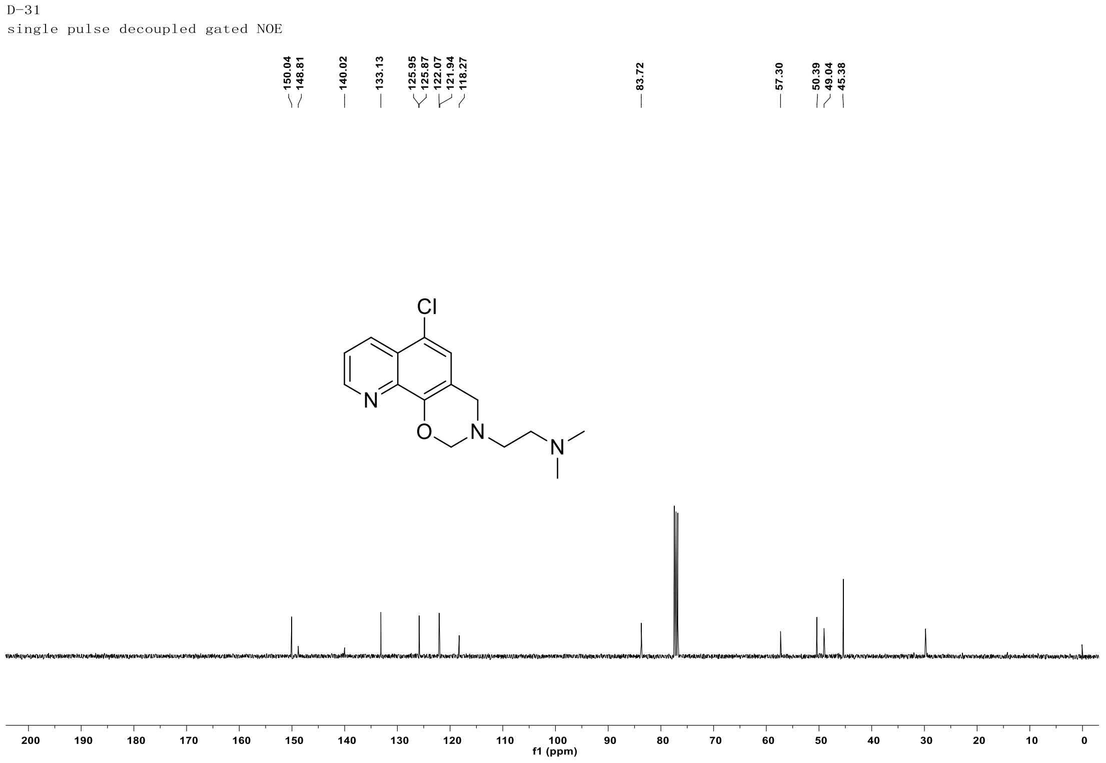

*Figure S7. ^{1}H NMR (top) and ^{13}C NMR (bottom) of 5-Chloro-13-cyanoethyl-3, 4-dihydro-2H-[1, 3] oxazino[5, 6-h]quinoline (**4g**)*

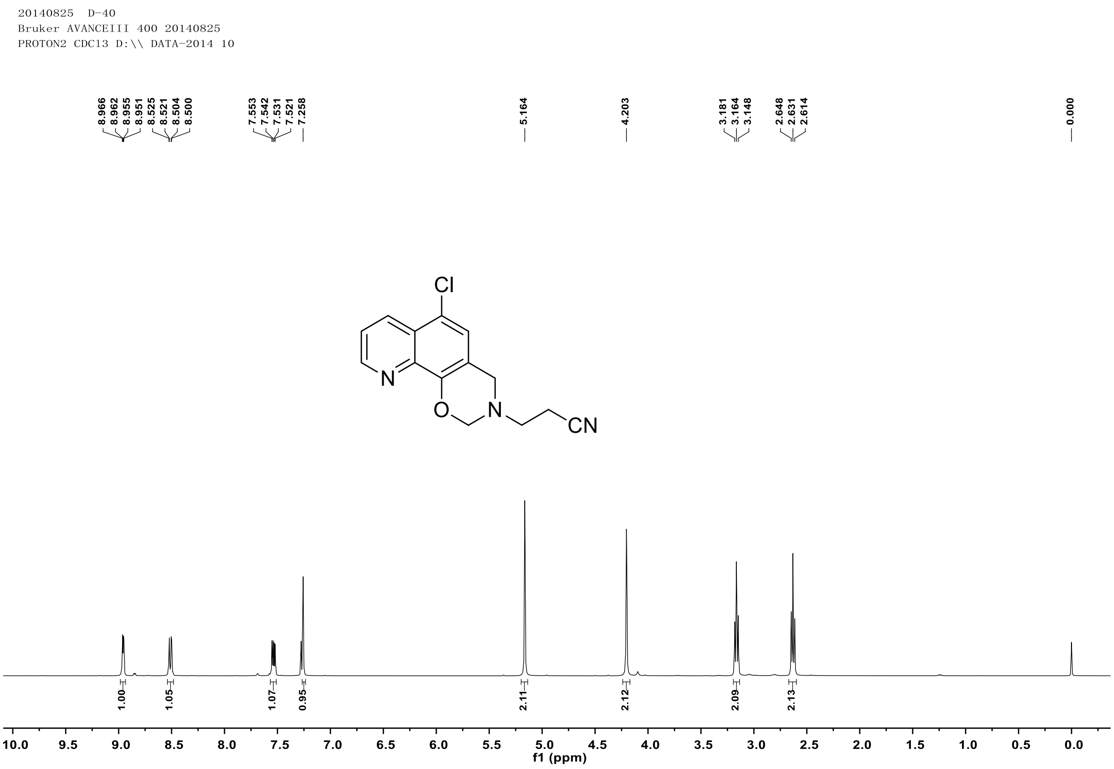

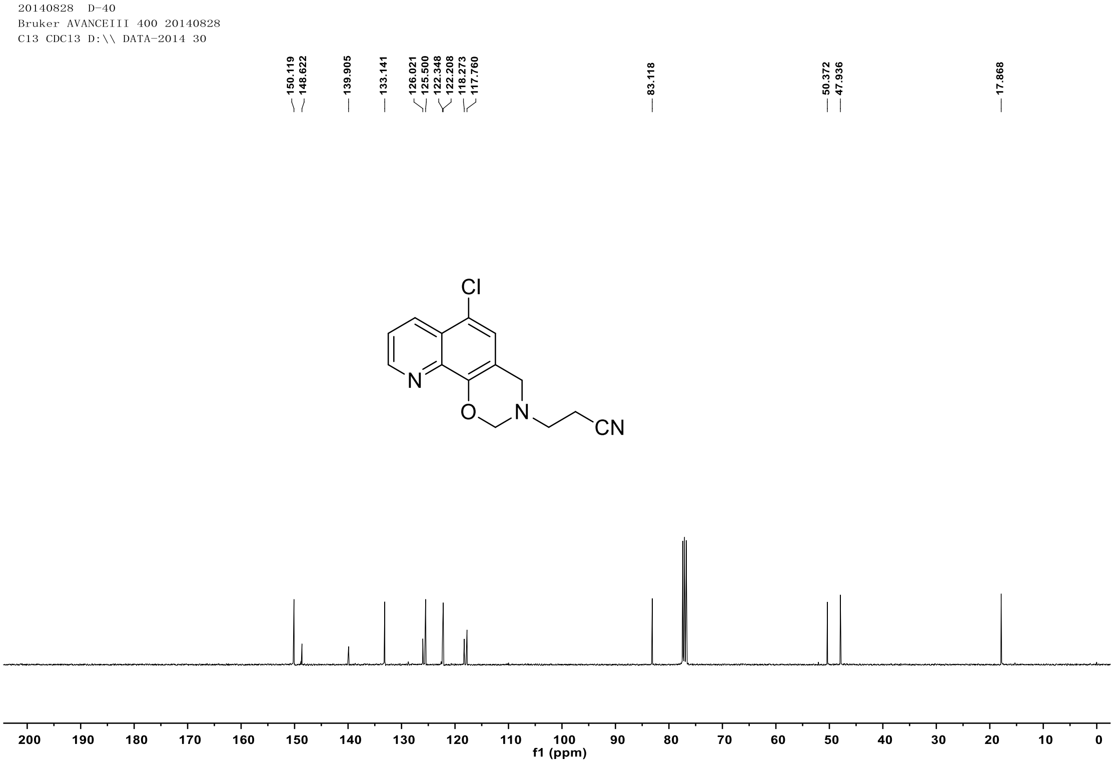

*Figure S8. ¹H NMR (top) and ¹³C NMR (bottom) of 5-Chloro-13-methoxycarbonylmethyl-3,4-dihydro-2H-[1,3]oxazino[5,6-h]quinoline (**4h**)*

20140708-D-24
BRUKER AV-III-500 1H-NMR D-24 IN DMSO 2014.07.08
PROTON DMSO E:\\ songdanqing 39

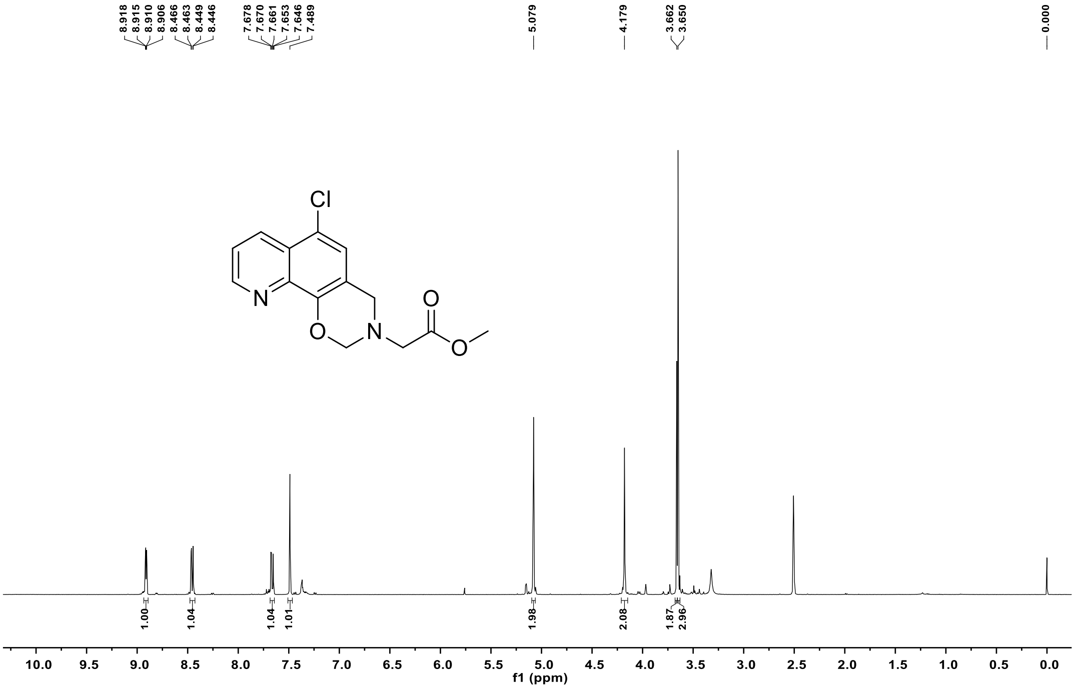

20140709-D-24
BRUKER AV-III-500 13C-NMR D-24 IN DMSO 2014.07.09
C13CPD DMSO E:\\ songdanqing 58

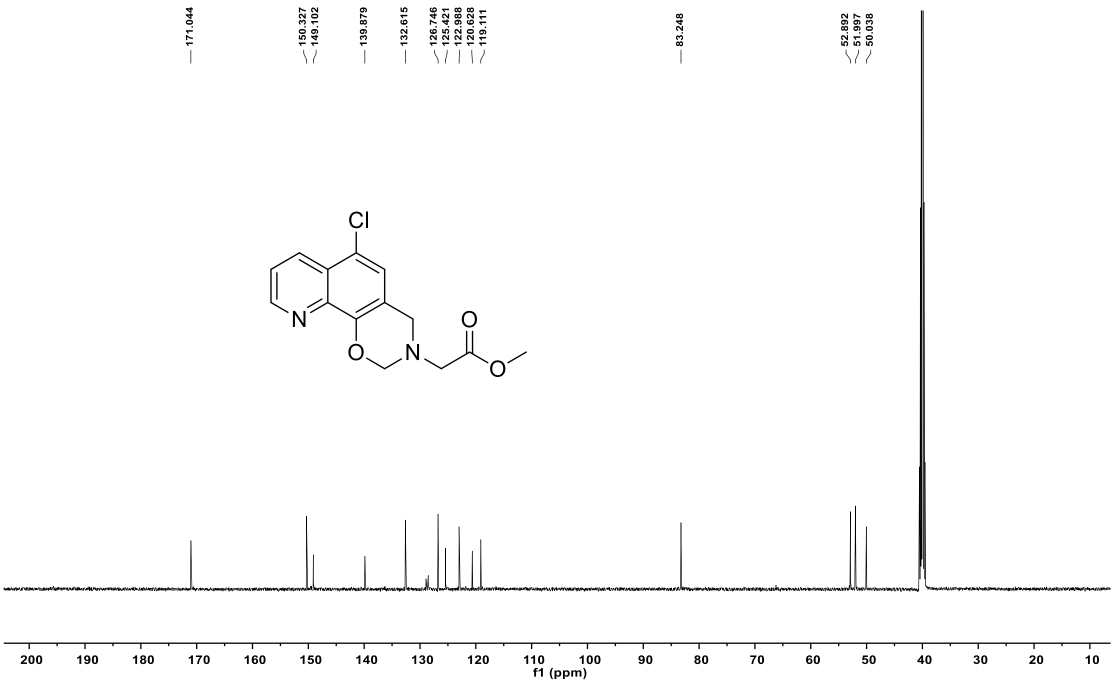

*Figure S9. ^{1}H NMR (top) and ^{13}C NMR (bottom) of 5-Chloro-13-benzoxycarbonylmethyl-3,4-dihydro-2H-[1,3]oxazino[5,6-h]quinoline (**4i**)*

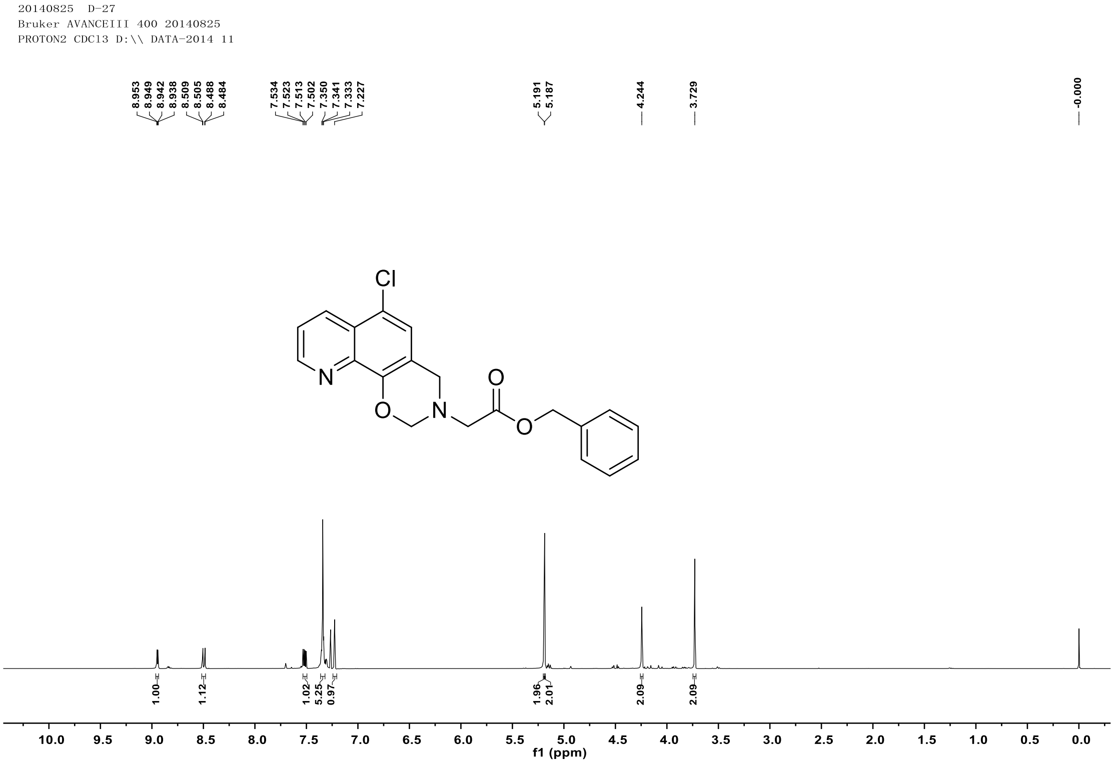

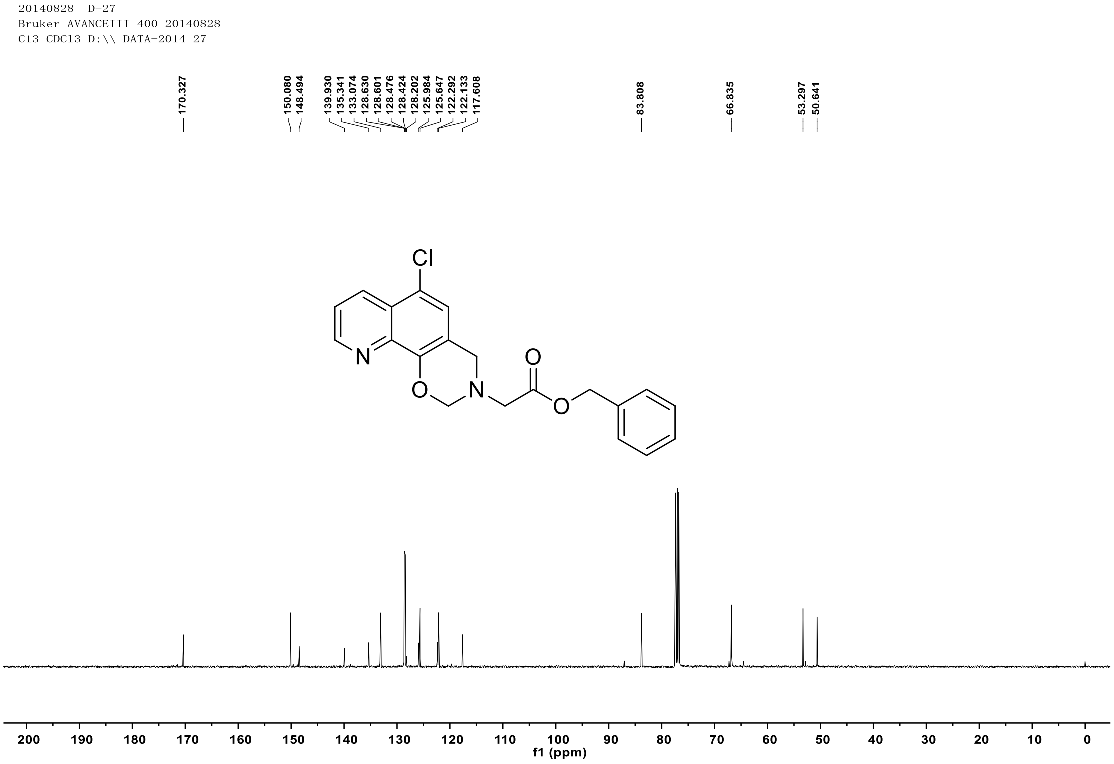

*Figure S10. ^{1}H NMR (top) and ^{13}C NMR (bottom) of 5-Chloro-13-cyclopropyl-3,4-dihydro-2H-[1,3]oxazino[5,6-h]quinoline (**4j**)*

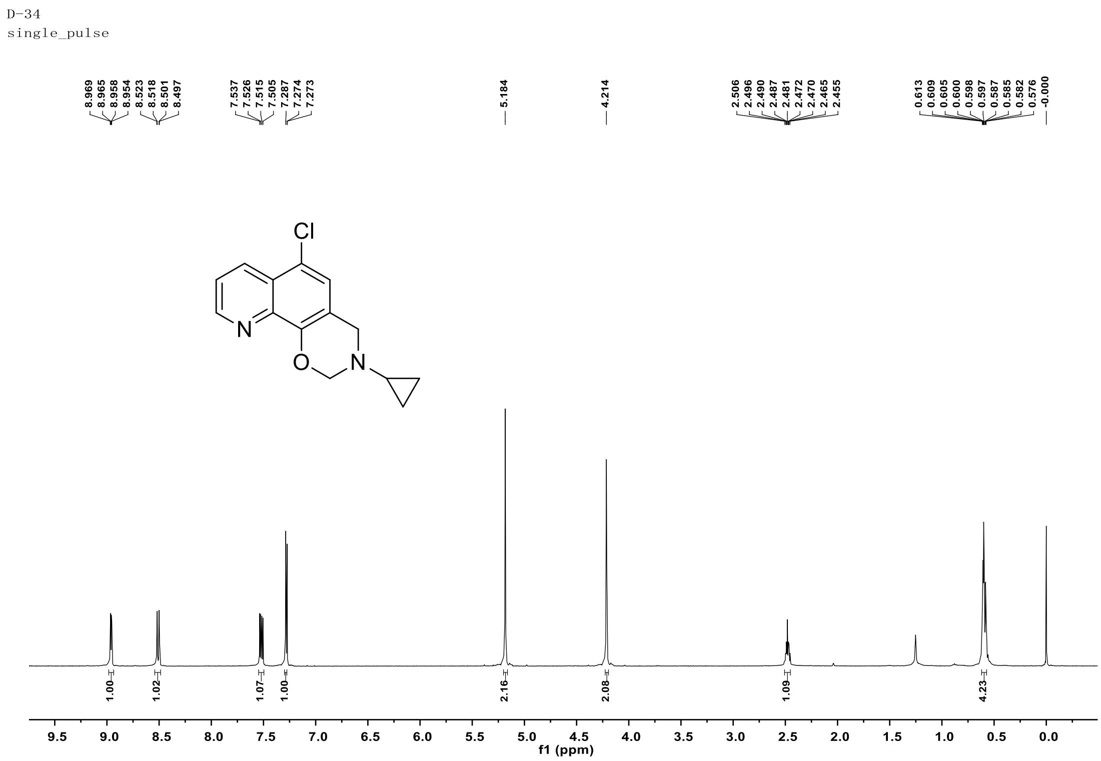

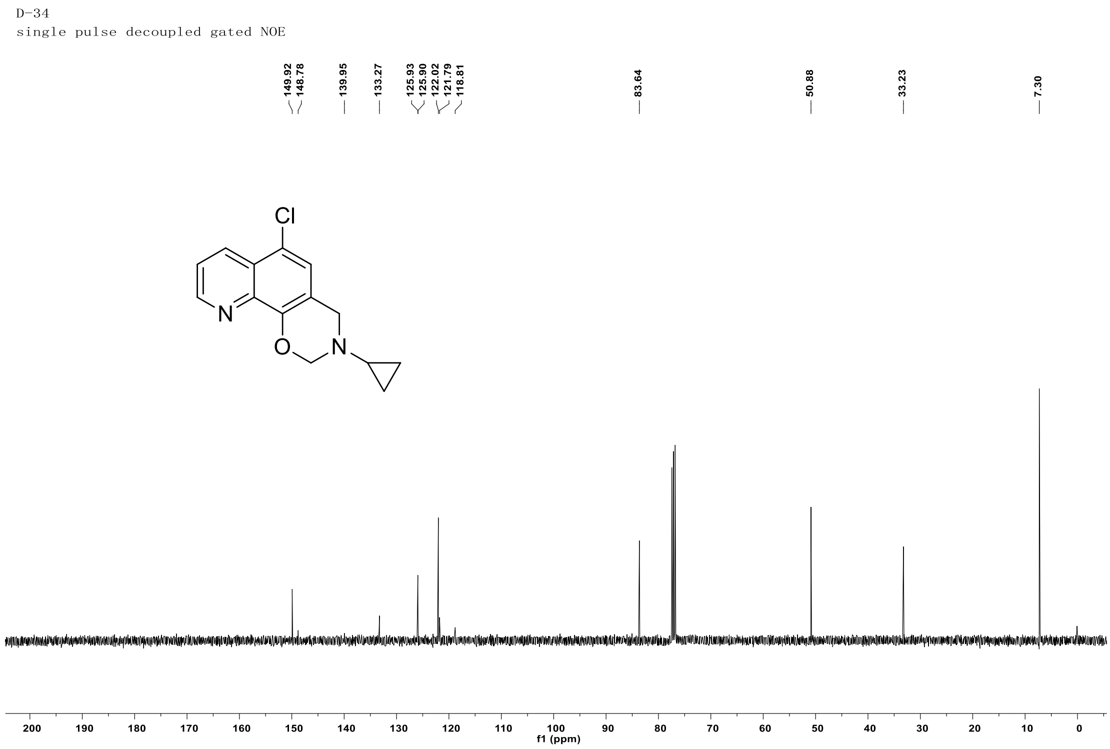

*Figure S11. ^{1}H NMR (top) and ^{13}C NMR (bottom) of 5-Chloro-13-[2-(pyrrolidin-1-yl)ethyl]-3,4-dihydro- 2H-[1,3]oxazino[5,6-h]quinoline (**4k**)*

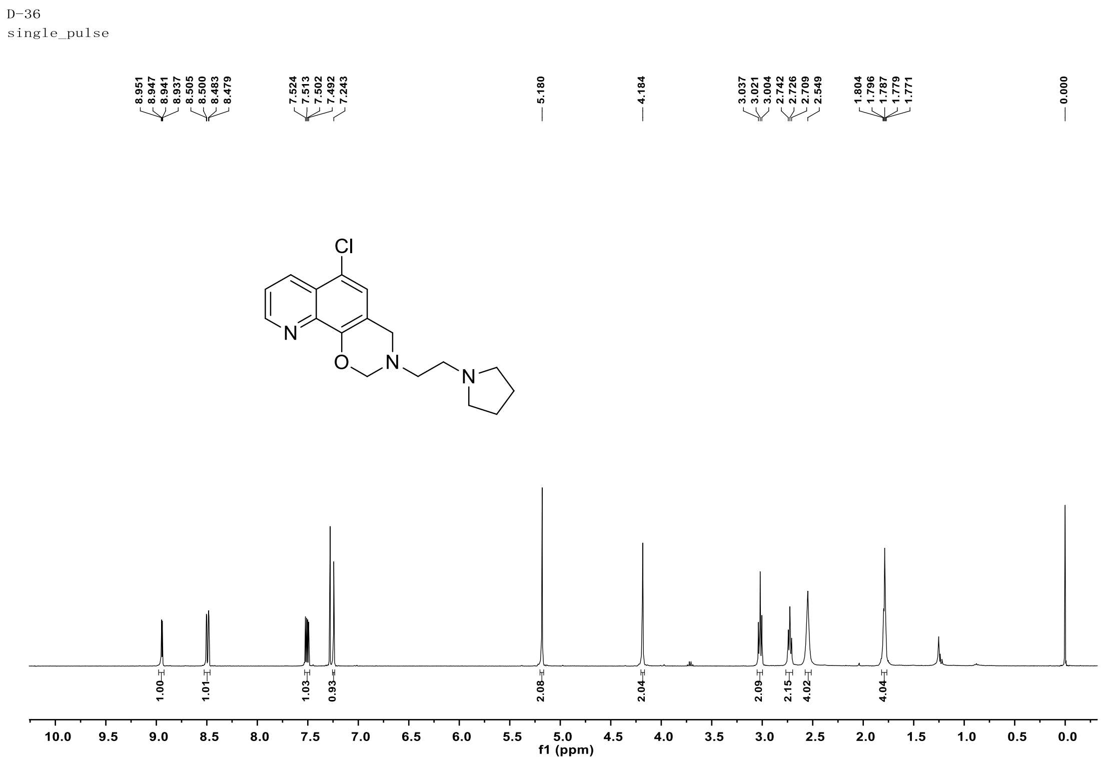

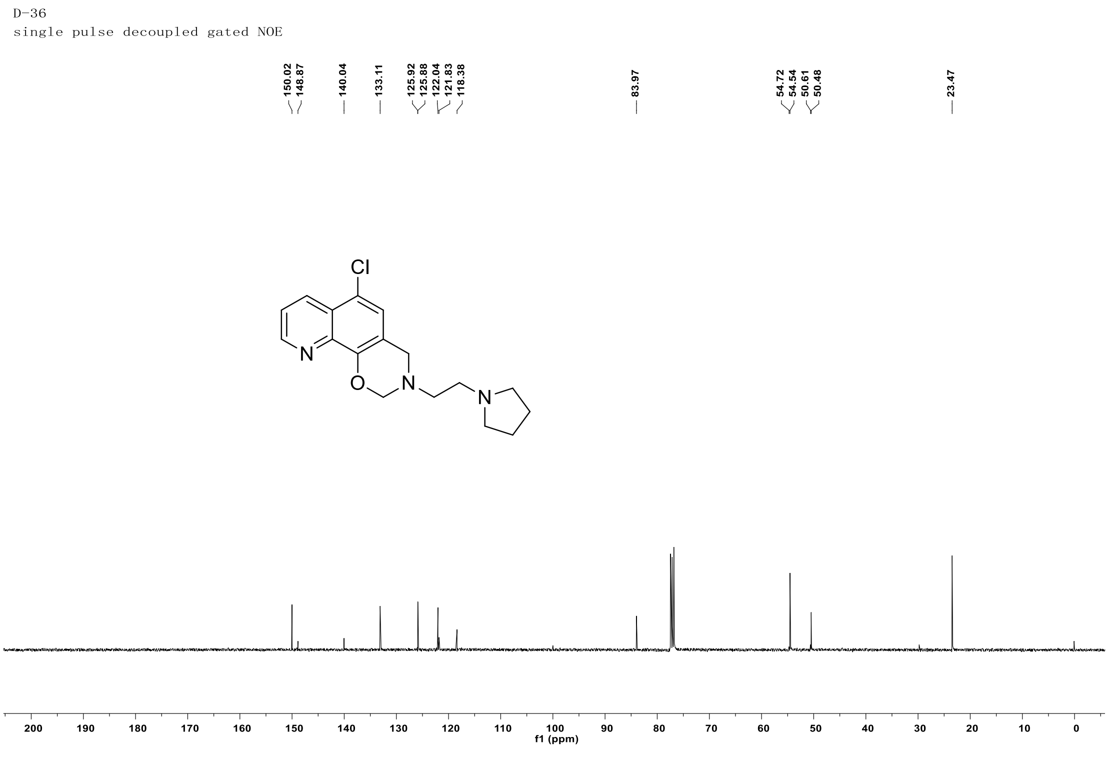

*Figure S12. ^{1}H NMR (top) and ^{13}C NMR (bottom) of 5-Chloro-13-[(tetrahydro-2H-pyran-4-yl)methyl]-3,4-dihydro-2H-[1,3]oxazino[5,6-h]quinoline (**4l**)*

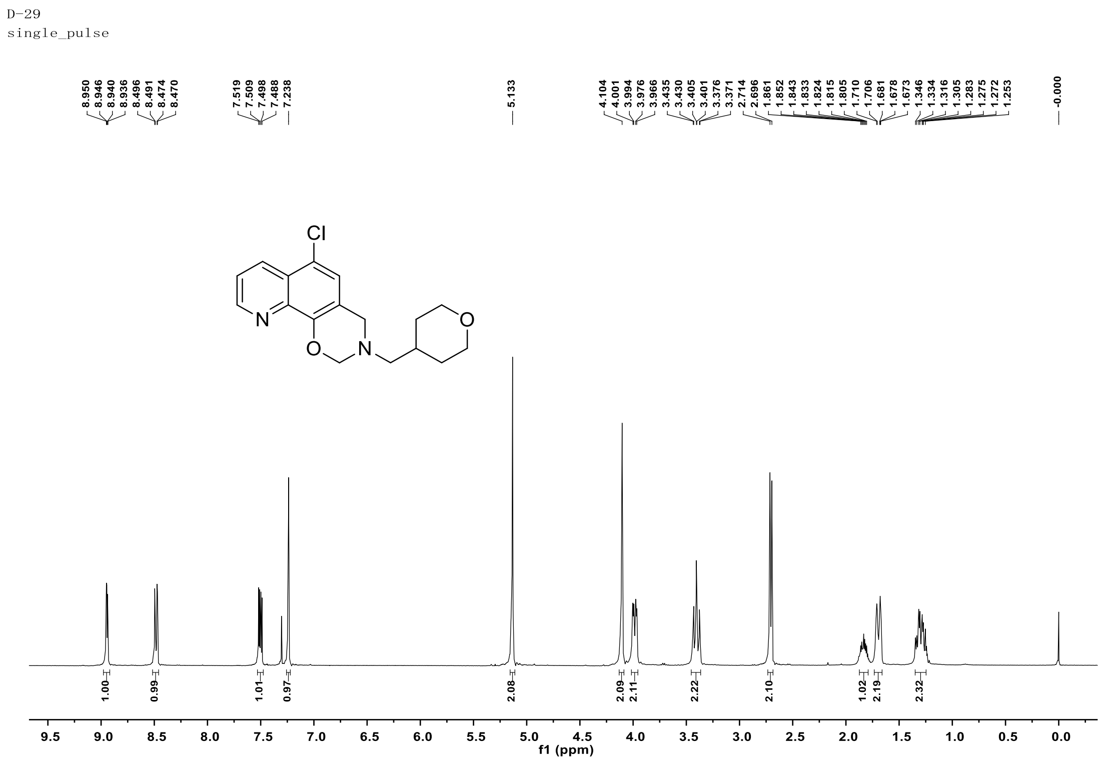

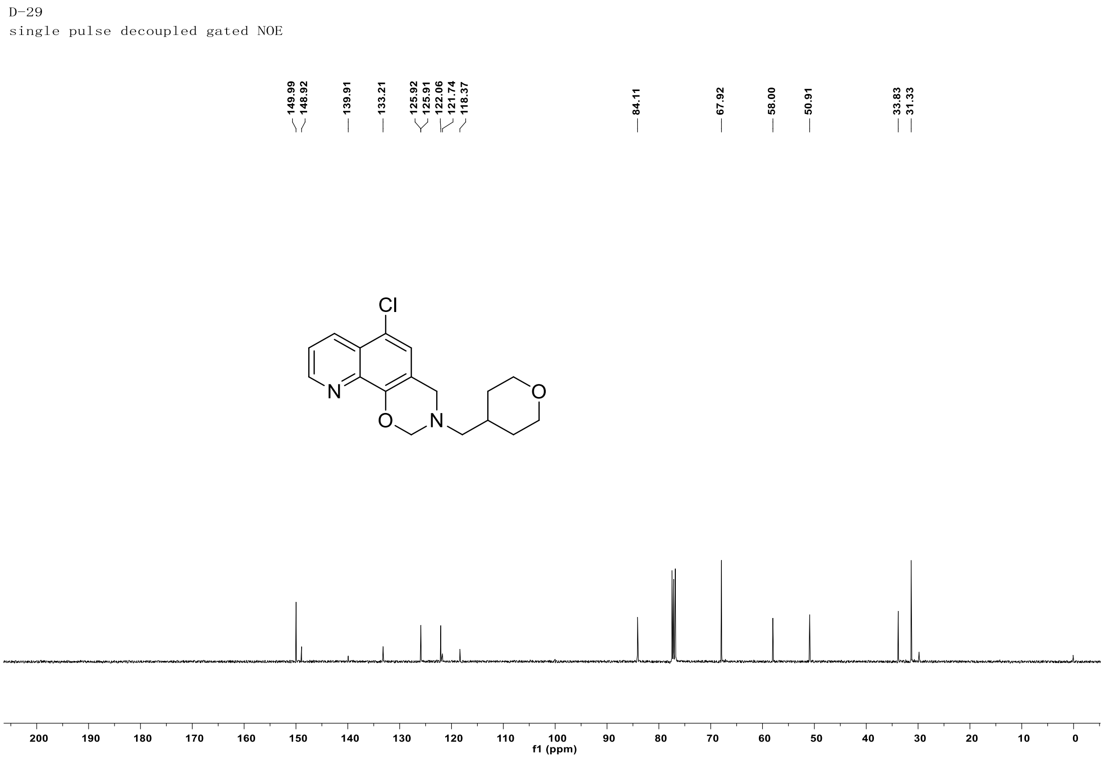

*Figure S13. ^{1}H NMR (top) and ^{13}C NMR (bottom) of 5-Chloro-13-morpholinopropyl-3,4-dihydro-2H-[1,3]oxazino[5,6-h]quinoline (**4m**)*

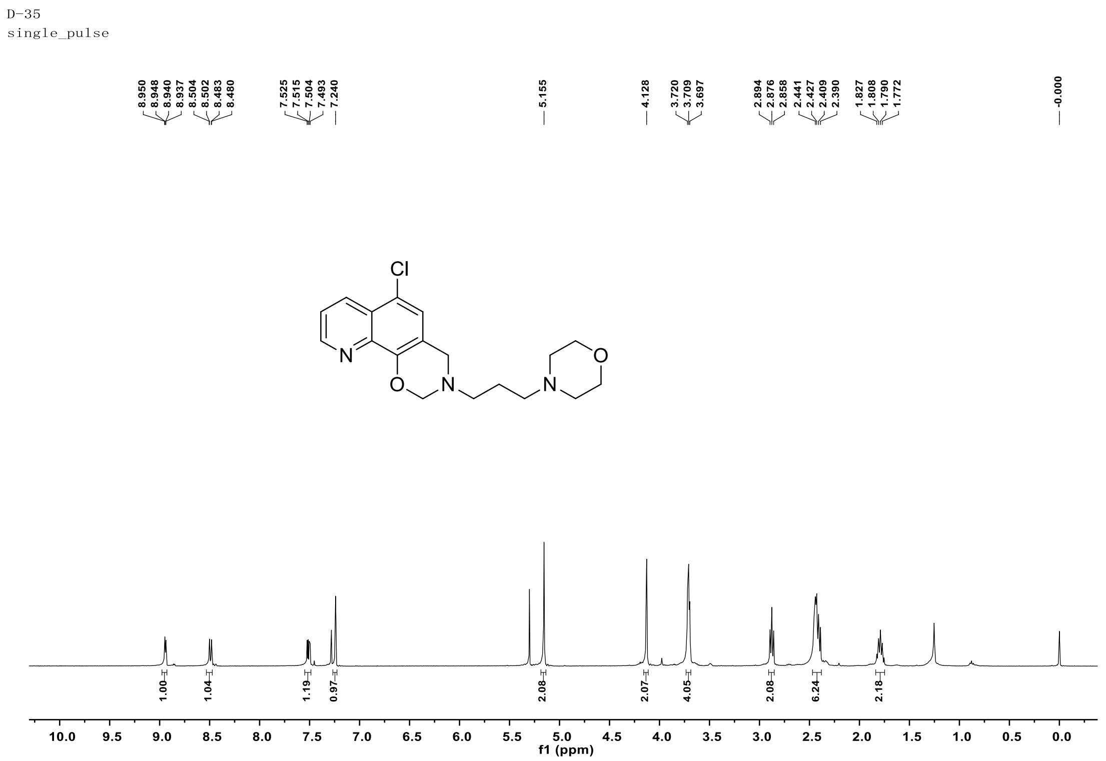

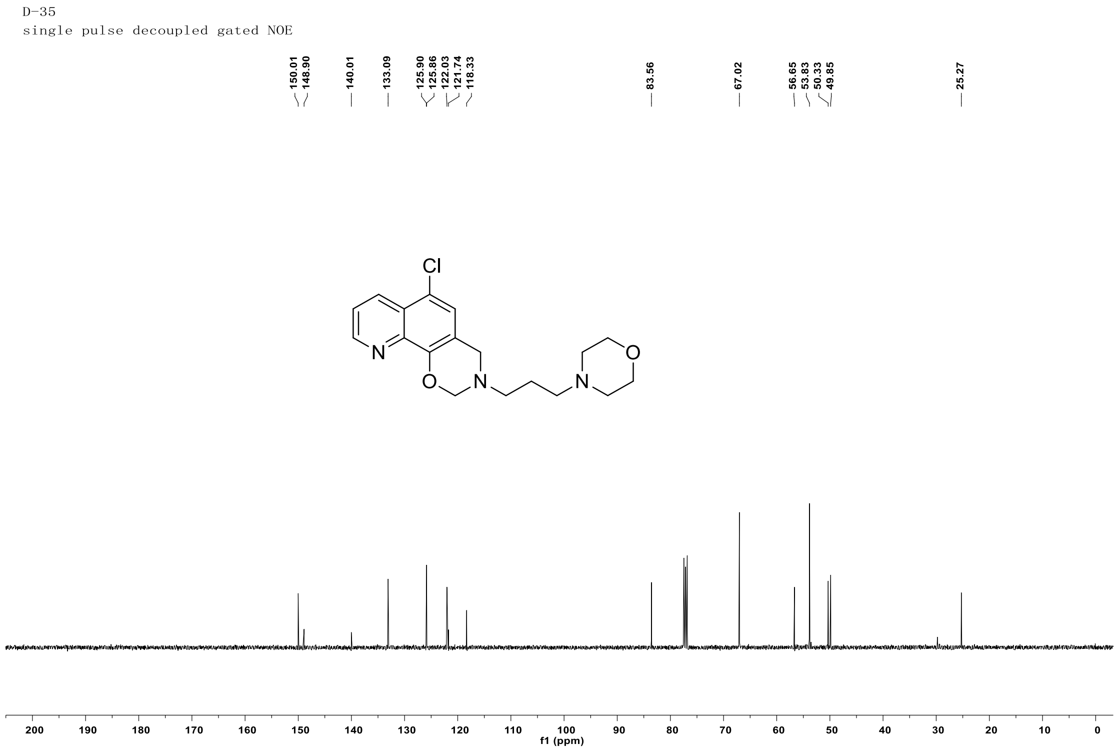

Figure S14. ^{1}H NMR (top) and ^{13}C NMR (bottom) of 5-Chloro-7-(pyrrolidin-1-yl-methyl)quinolin-8-ol ***(5a)***

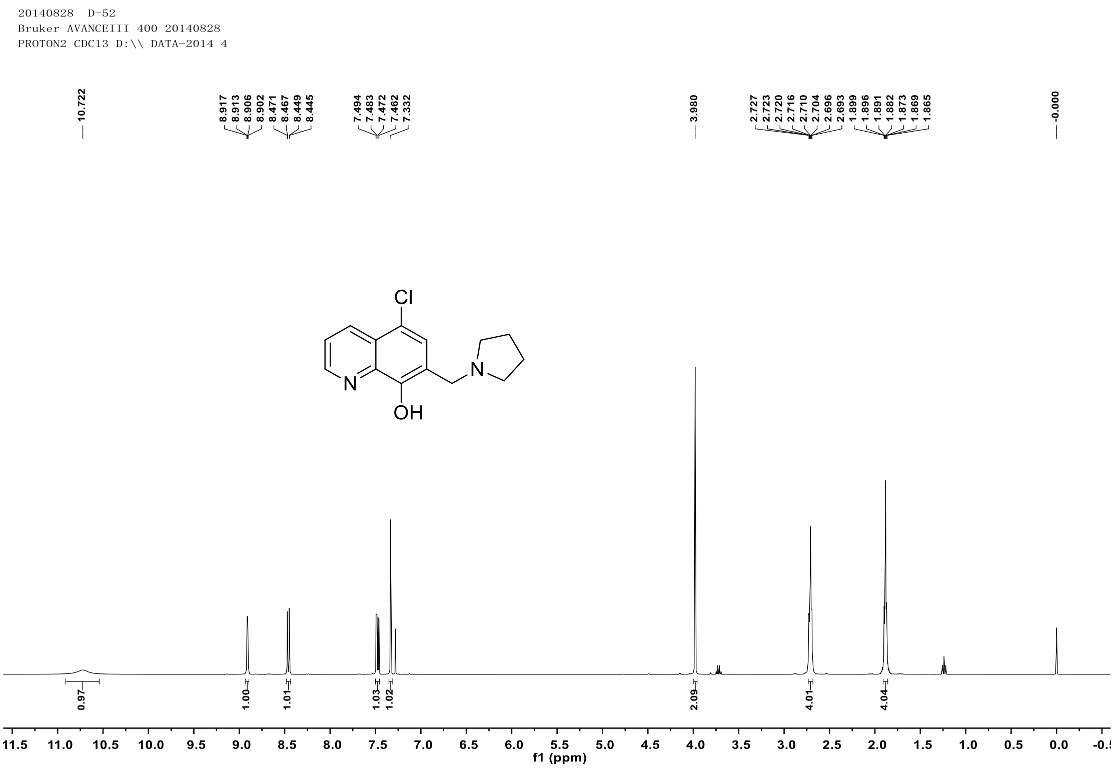

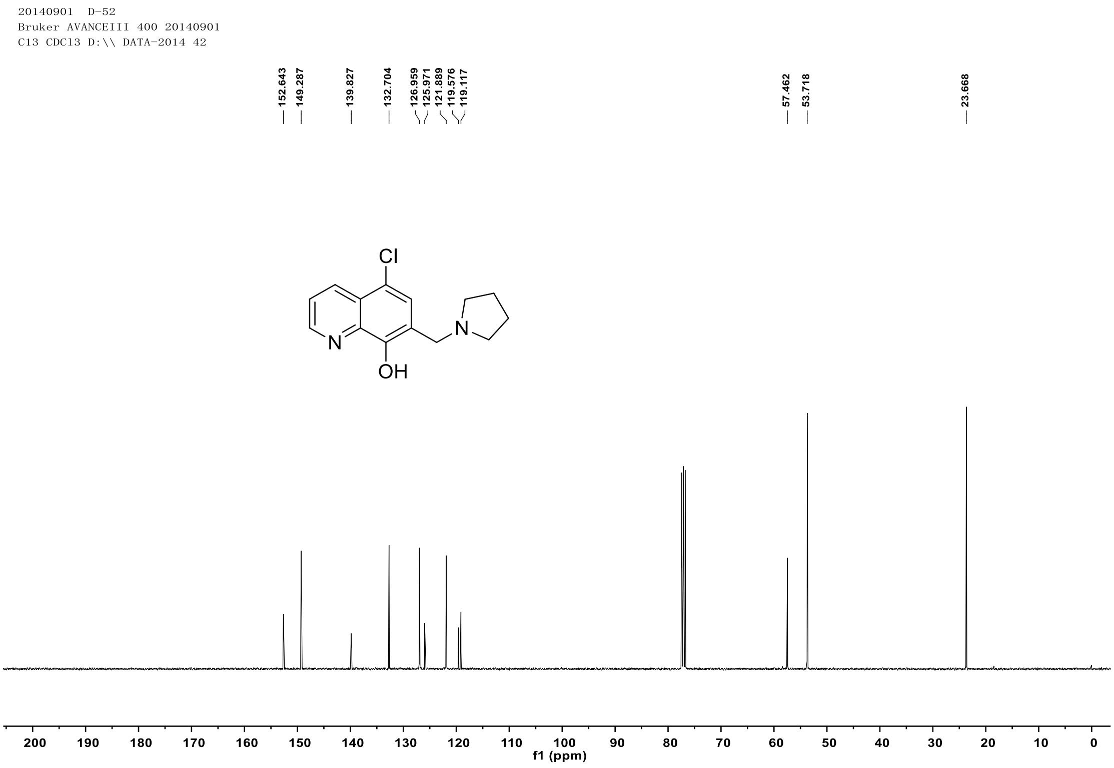

*Figure S15. ^{1}H NMR (top) and ^{13}C NMR (bottom) of 5-Chloro-7-(morpholinomethyl)quinolin-8-ol (**5b**)*

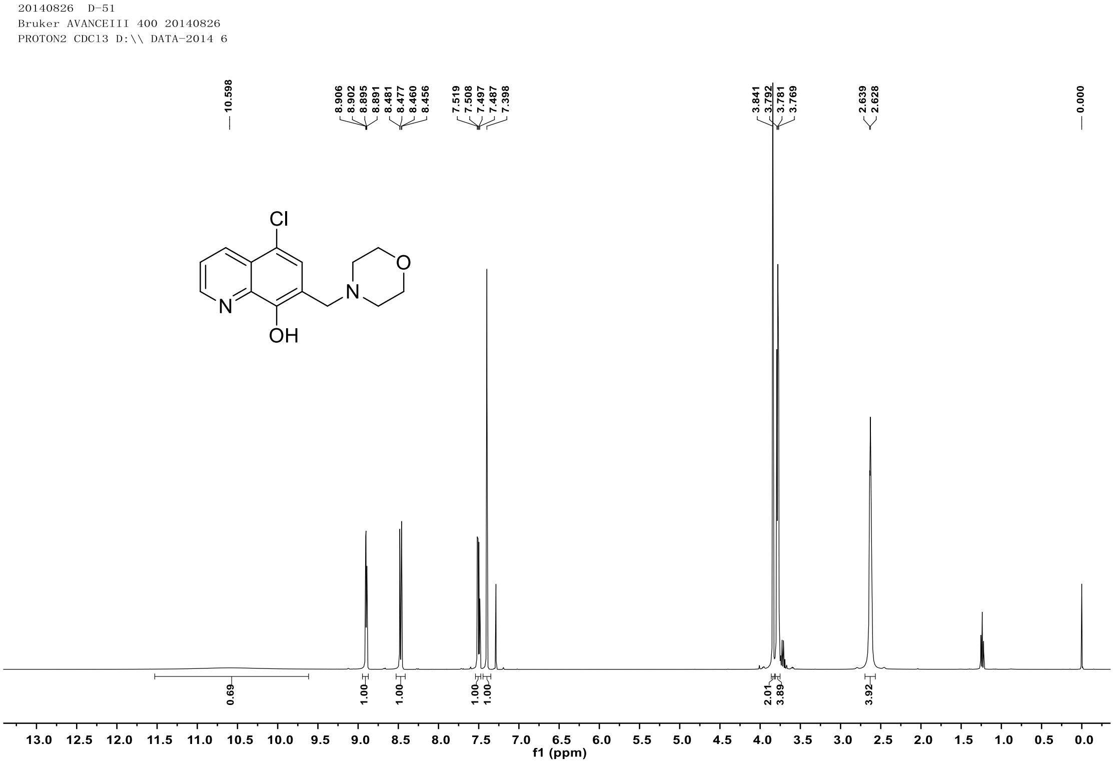

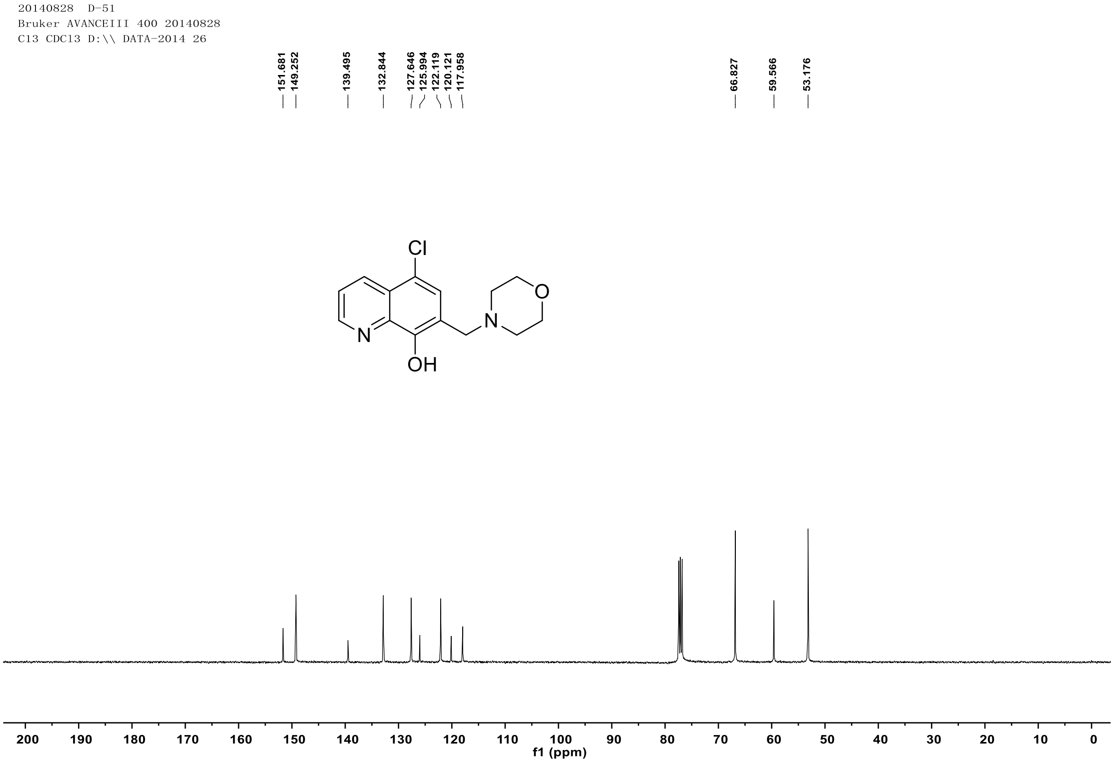

*Figure S16. ^{1}H NMR (top) and ^{13}C NMR (bottom) of 5-Chloro-7-[(4-methylpiperazin-1-yl)methyl] quinolin-8-ol (**5c**)*

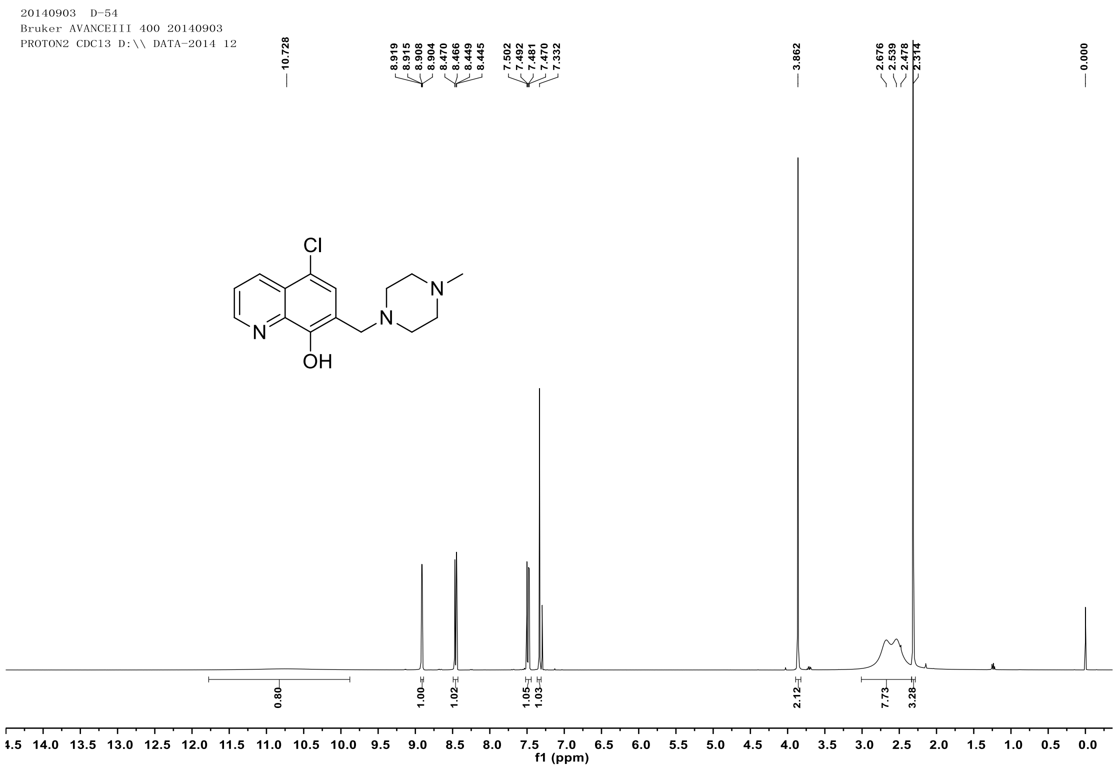

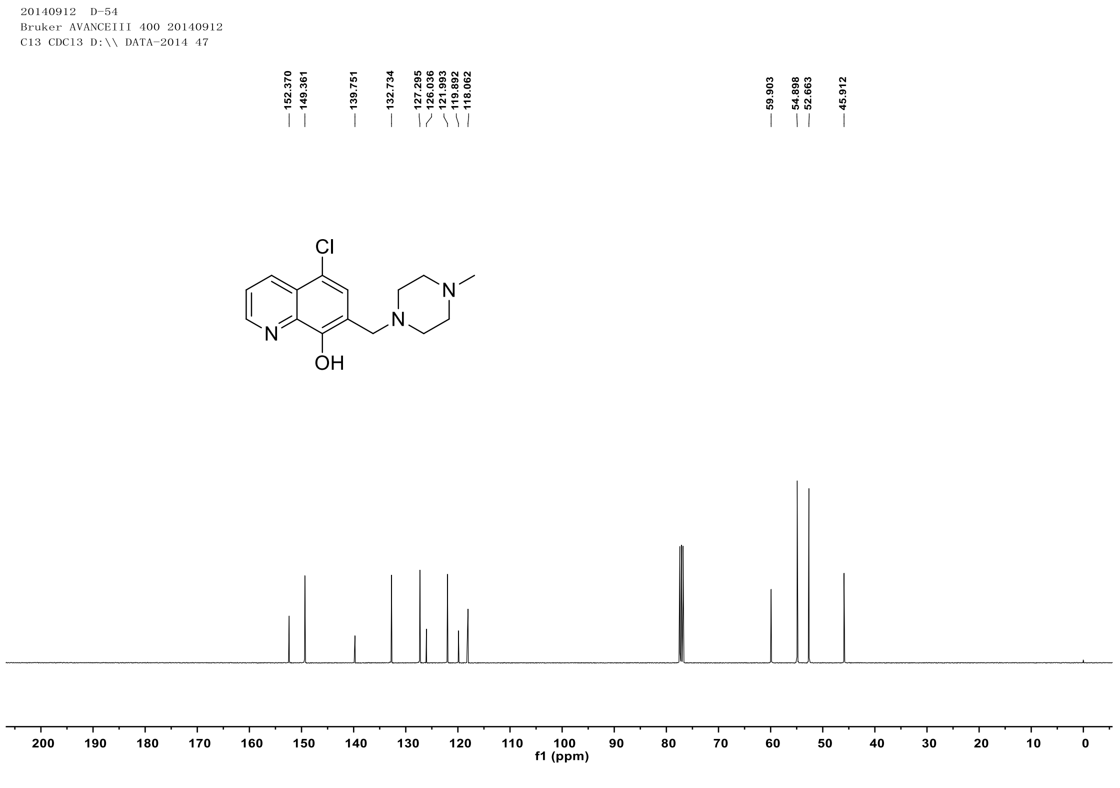

Figure S17. ^{1}H NMR (top) and ^{13}C NMR (bottom) of 7-{4-[(5-chloro-8-hydroxyquinolin-7-yl)methyl] piperazin-1-yl}-1-cyclopropyl-6-fluoro-4-oxo-1,4-dihydro-1,8-naphthyridine-3-carboxylic acid ***(5d)***

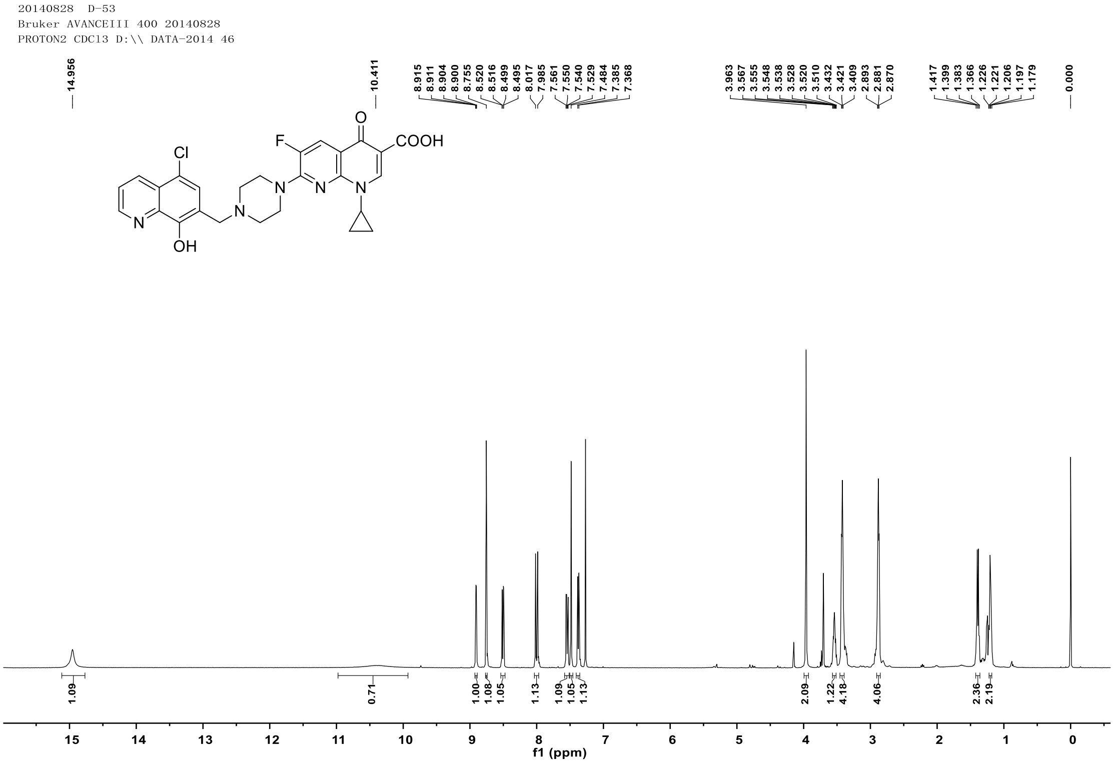

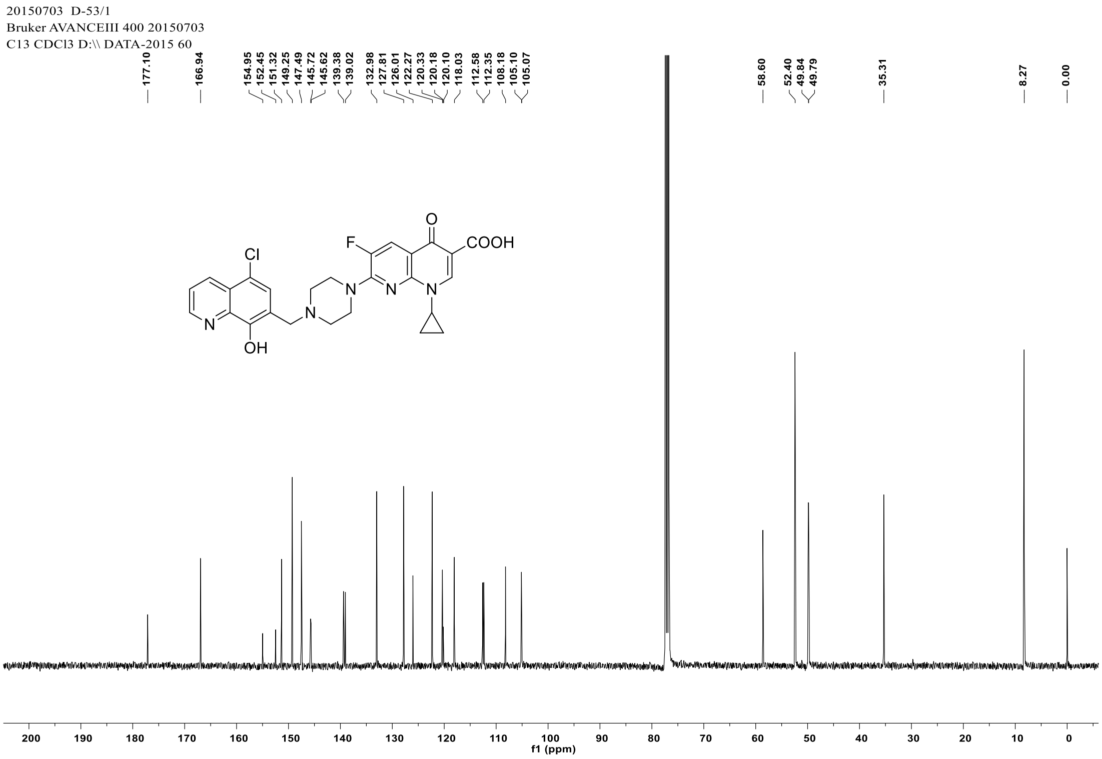

*Figure S18. ^{1}H NMR (top) and ^{13}C NMR (bottom) of 7,7'-[[(3-ethoxypropyl)azanediyl]bis(methylene)] bis(5-chloroquinolin-8-ol) (**6a**)*

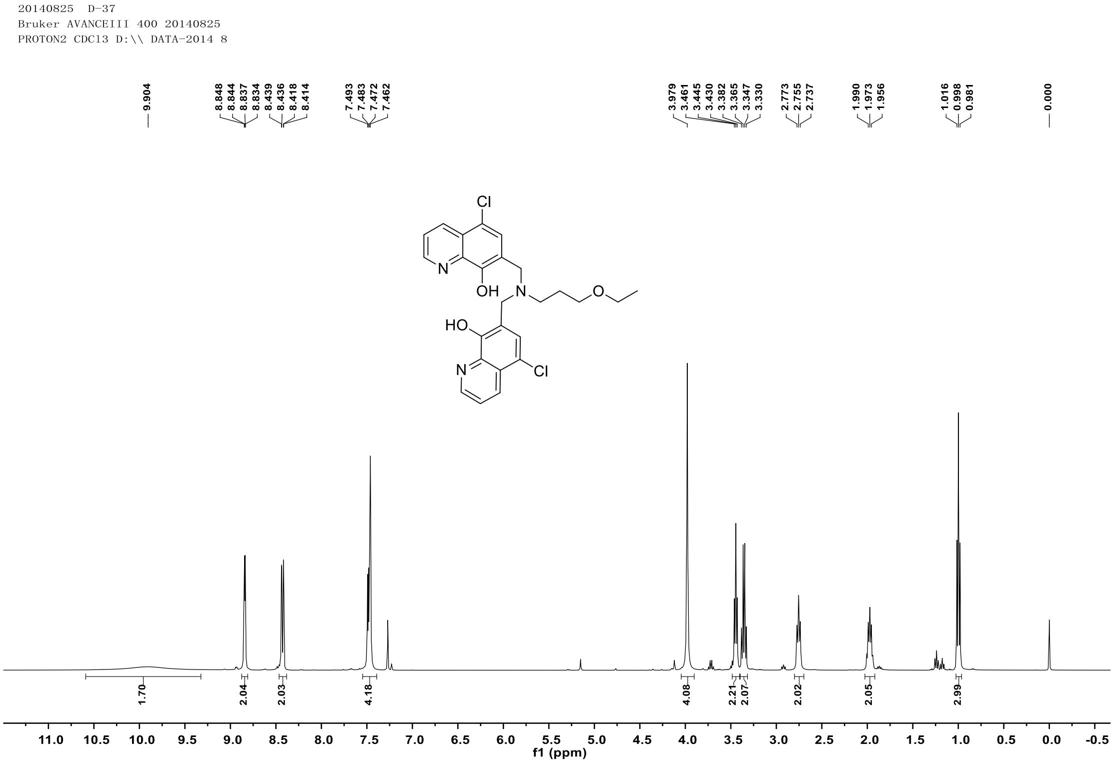

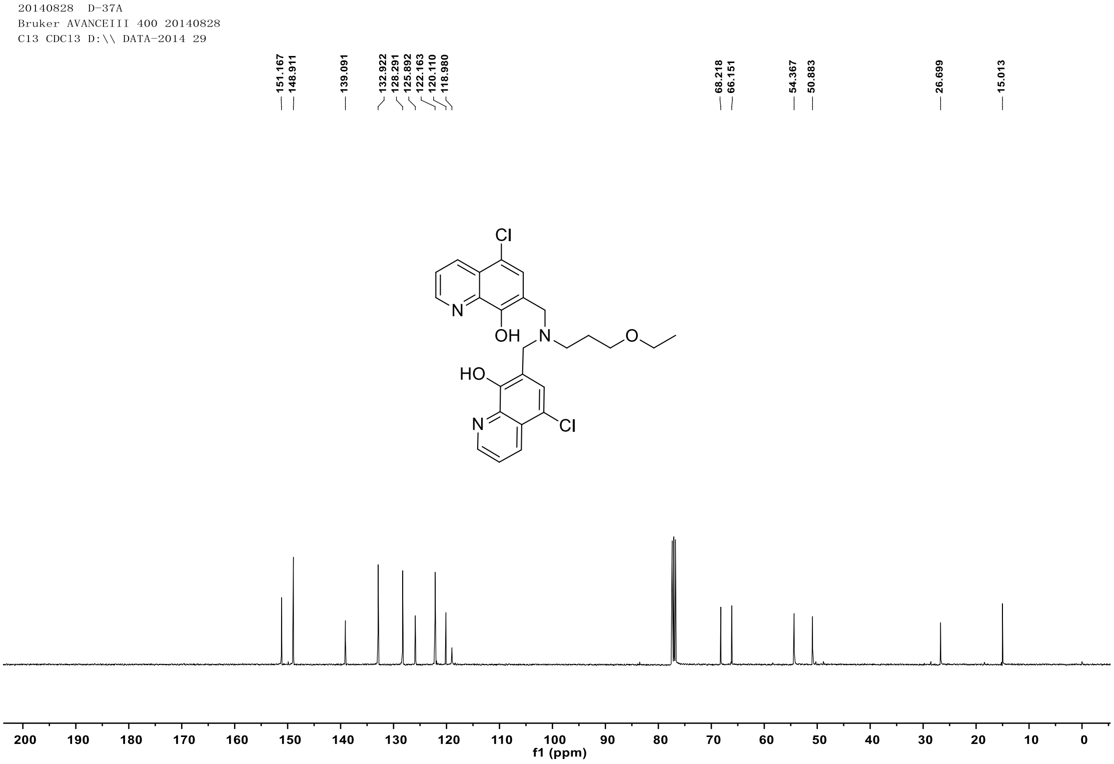

*Figure S19. ^{1}H NMR (top) and ^{13}C NMR (bottom) of (S)-ethyl-2-[bis[(5-chloro-8-hydroxyquinolin-7-yl)methyl]amino]-3-phenylpropanoate (**6b**)*

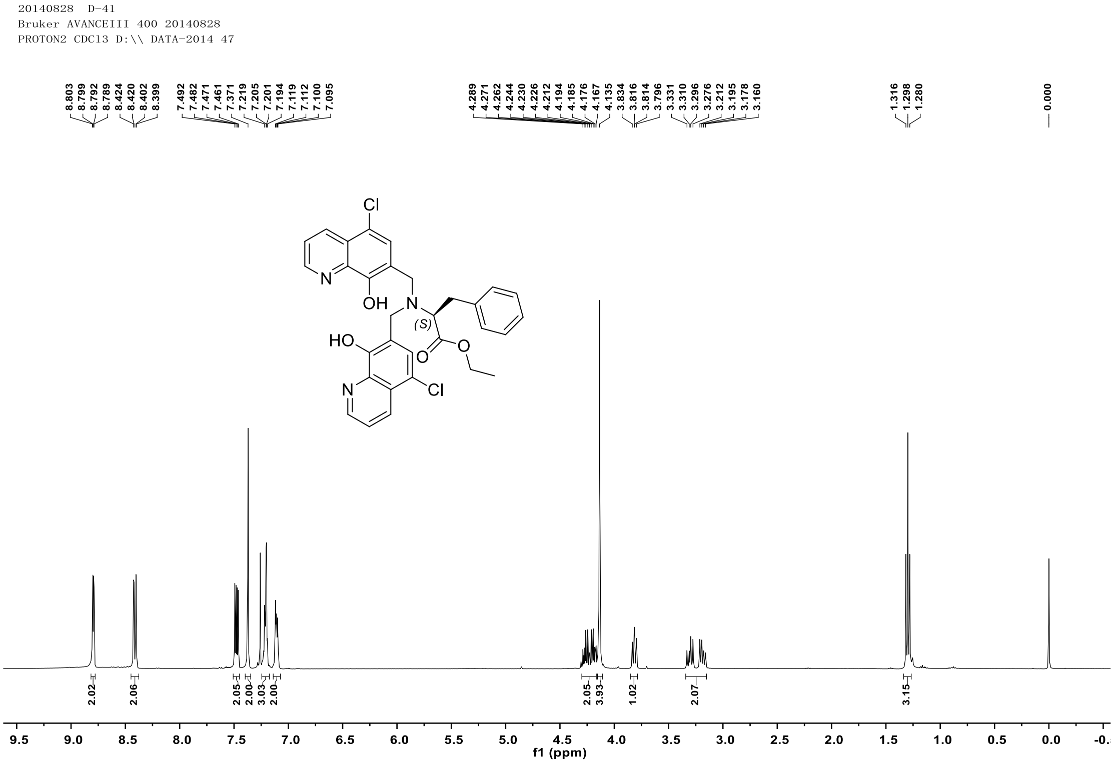

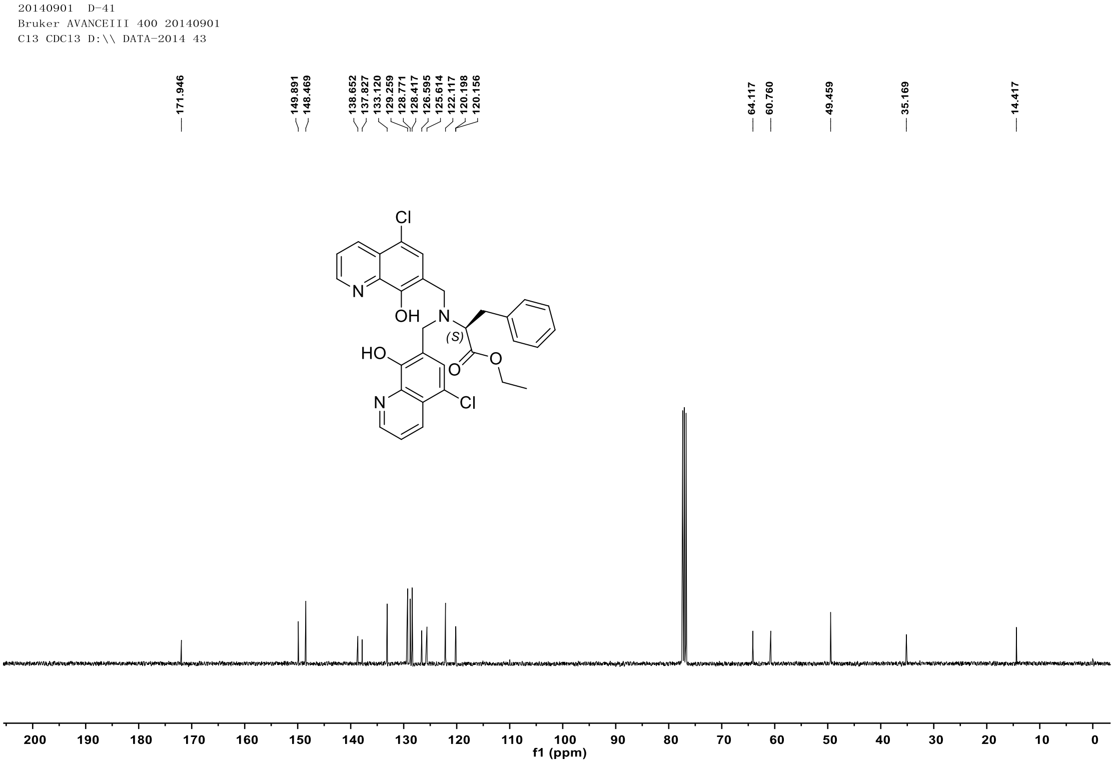

Supplement: Supplementary file 1 [file molecules-24-00548-s001.pdf]
